# Minimizing Delay and Transmission Times with Long Lifetime in Code Dissemination Scheme for High Loss Ratio and Low Duty Cycle Wireless Sensor Networks

**DOI:** 10.3390/s18103516

**Published:** 2018-10-18

**Authors:** Wei Qi, Wei Liu, Xuxun Liu, Anfeng Liu, Tian Wang, Neal N Xiong, Zhiping Cai

**Affiliations:** 1 School of Information Science and Engineering, Central South University, Changsha 410083, China; weiqicsu@csu.edu.cn; 2 School of Informatics, Hunan University of Chinese Medicine, Changsha 410208, China; weiliu@csu.edu.cn; 3 College of Electronic and Information Engineering, South China University of Technology, Guangzhou 510641, China; liuxuxun@scut.edu.cn; 4 The State Key Laboratory of Industrial Control Technology, Zhejiang University, Hangzhou 310027, China; 5 College of Computer Science and Technology, Huaqiao University, Xiamen 361021, China; wangtian@hqu.edu.cn; 6 Department of Mathematics and Computer Science, Northeastern State University, Tahlequah, OK 74464, USA; xiongnaixue@gmail.com; 7 Department of Networks Engineering, School of Computer, National University of Defense Technology, Changsha 410073, China; zpcai@nudt.edu.cn

**Keywords:** wireless sensor networks, code dissemination, delay, lifetime, transmission times, low duty cycle

## Abstract

Software defined networks brings greater flexibility to networks and therefore generates new vitality. Thanks to the ability to update soft code to sensor nodes, wireless sensor networks (WSNs) brings profound changes to Internet of Things. However, it is a challenging issue to minimize delay and transmission times and maintain long lifetime when broadcasting data packets in high loss ratio and low duty cycle WSNs. Although there have been some research concerning code dissemination, those schemes can only achieve a tradeoff between different performances, instead of optimizing all these important performances at the same time. Therefore, in this paper we propose a new strategy that can reduce delay and transmission times simultaneously. In traditional method, the broadcasting nature of wireless communication is not sufficiently utilized. By allowing sons of the same parent node to share awake slots, the broadcasting nature is well exploited and delay is thus reduced as well as transmission times with lifetime not affected. And, as we discover there is energy surplus when collecting data in area away from sink, we further improve this strategy so that all the performances can be further bettered. Compared with traditional method, the methods we design (IFAS, BTAS and AAPS) can respectively reduce delay by 20.56%, 31.59%, 55.16% and reduce transmission times by 29.53%, 43.93%, 42.04%, while not reducing lifetime.

## 1. Introduction

Wireless sensor networks (WSNs), consisting of tiny wireless sensing devices equipped with data processing and communication capabilities, is an important element for realizing the Internet of Things (IoT) [1,2,3,4,5]. And, Software defined networks (SDNs) brings more application prospect to WSNs [6,7]. With SDNs, sensor nodes can obtain new features and expand their application domain by merely receiving new soft code without redeployment. Therefore, SDNs plays a key role in reducing deployment costs and improving networks performance [8,9,10,11,12].

However, SDNs also brings new challenges to WSNs [13,14,15,16]. In WSNs, the sink node receives new soft code first, then broadcasts the code in the networks by radio. Therefore, the nodes receiving the new soft code can obtain new functions and generate new applications [7,13,15]. Through SDNs, the function and application of networks can be redefined by merely changing the node’s code [13,15]. As a result, the deployment costs are greatly reduced and the procedure of promoting new features is accelerated, which adapts to the rapid development of Internet of Things [17,18,19,20,21]. However, one of the most challenging issues is how to disseminate soft code to every node effectively [6,7,11,13,15]. There are mainly three performance indicators to measure the spread of code: (1) delay; (2) lifetime; (3) transmission times.

(1) The delay of dissemination refers to the time gap between the moment when sink node begins to transmit code and the moment when the last node in the networks succeeds in receiving the code [6,7,11,13,15]. Obviously, application requires the delay to be as small as possible. For instance, in the monitoring and data perception in spot of industry, if data collection frequency, calculation method or type of data perceived needs changing, these new features can be realized by updating the sensor node’s code [21,22,23,24]. Apparently, during the update process, there will be difference between the nodes with new code and others without it, which will result in some inconsistency between the old and new systems. It is evident that the duration of such inconsistency should be as short as possible, so that all the nodes can quickly adopt the new system. Thus, the delay of dissemination should be as small as possible. 

(2) The lifetime of networks usually refers to how long it functions before the first node dies. Obviously, it is required that lifetime should be as long as possible. However, one of the key characteristics of sensor node is that it is usually powered by battery and due to the constraints of manufacturing costs, it should be as small as possible, which also makes it easier for nodes to be deployed [25,26,27]. Under this circumstance, the volume of the batteries is small as well. Also, much research suggests that the WSN is usually deployed in dangerous places [28,29,30,31], or places inaccessible to human beings, which renders it unrealistic to change the dead batteries for new ones; this is another challenging issue to save node energy for longer lifetime [32,33,34,35]. In WSNs, one of the effective ways to save energy is the scheme of duty cycle [7,15]. In this scheme, sensor nodes adopt the method of periodical sleep/awake. Since the node in sleep mode consumes less than one percent of the energy consumed in awake mode, the node should be set in sleep mode as frequently as possible [7,15]. In realistic duty cycle based WSNs, time is divided into several homogenous slots and during one slot, node can complete the receiving and sending of a packet [7,15]. Thus, the duty cycle should be set relatively small for the networks with sparse data. A duty cycle consists of *n* slots and the duration of one cycle is T, during which there is only one awake slot and the other *n* − 1 slots are in sleep mode. Although the nodes can save energy by adopting the duty cycle, it causes greater delay. That is because when sender is ready to broadcast the code, it must wait until receiver is awake before beginning to transmit packet.

(3) Reduce transmission times. In addition, radio is by nature capable of broadcasting. Namely, when the sender is transmitting data, it is possible for all the nodes within its sending radius to receive it [36,37,38,39,40]. Therefore, by exploiting this nature of wireless broadcasting, transmission times can be greatly reduced [41,42,43,44]. Transmission times can directly affect the energy consumption of node [45,46,47]. Researchers suggest the greatest energy-consuming operation of sensor nodes is receiving and sending data, which takes up over 80% of the energy consumption. Therefore, during propagation, since the size of the code is fixed, it can be divided into N data packets to be sent. If the transmission times of one packet can be effectively reduced, it will naturally reduce transmission times required for the entire code propagation. Thus, to reduce the number of packets sent by nodes is the key to reduce energy consumption.

But, to reduce the number of packets sent is not an easy task. First of all, since nodes select slot randomly in low duty cycle WSNs [7,14,15], when the sender is transmitting data packet, there is not many nodes awake within its sending radius, which means the broadcasting nature cannot be fully utilized. What is worse, because of the influence of communication channel and communication environment, there is a certain loss ratio [48,49]. In hostile WSNs, the loss ratio can be over 20% [50]. In order to guarantee high reliability of data communication in the environment of unreliable communication, the general method is retransmission mechanism: when transmission fails, sender uses re-sending or multi-resending to increase the reliability of transmission [50]. As a result, the unreliability of wireless channel increases the number of retransmissions and energy consumption is thus increased.

There have been some studies on code diffusion [6,7,11,13,15]. These studies generally abstract networks into a tree, in which the sink node is the root and the starting point of code diffusion. Parent nodes conduct data sending operation at their son nodes’ awake slots in turn. If a son node fails to receive data, parent node will send it again in the next cycle. And so on until the data is successfully received. The research in Ref. [51] points out: In reducing the times of data sending, the networks graph generated according to the minimum spanning tree method can achieve the best result. However, the delay in networks where the code is diffused along the minimum spanning tree is not necessarily the minimum, while it is the minimum when data transmission is conducted according to the networks graph generated along the shortest path from root to each node but its transmission times is not necessarily the smallest. Therefore, Ref. [51] proposed a method which can achieve tradeoff between delay and transmission times.

From the above discussion, it can be concluded that previous studies still have the following deficiencies: (1) The delay is comparatively large and in many studies, if the loss ratio of wireless link is p| 0≤p≤1, in terms of expected probability, the average transmission times is approximately $\;\bar{t}=1/p$. Namely, the expected delay of one hop is $\bar{\;D}=\bar{t}\times T=\left(1/p\right)\times T$. Obviously, we have $\bar{\;D}\geq T$. In other words, the expected delay of one hop in WSNs without loss should be less than a cycle T, while in WSNs with loss, 1/p T is required [51]. Therefore, for the networks with high loss rate, the delay is large, while the transmission from sink to the final node needs multiple hops, the delay of each hop accumulates to very large extent. (2) Excessive transmission times. As parent node broadcasts at every son node’s awake slot, it does not fully take advantage of the radio function of wireless transmission, so it does not effectively reduce the transmission times. If several son nodes are in awake mode while the parent node is transmitting, only one broadcast is required to send data to those nodes, which can effectively reduce transmission times. Reducing the transmission times can effectively improve the lifetime of networks. (3) Previous strategies are often unable to achieve optimization of delay, transmission times and lifetime at the same time. The reduction of delay is usually achieved at the price of shorter lifetime, or transmission times is reduced by enlarging delay, which is a tradeoff instead of all-round optimization.

Based on the above analysis, this paper proposes an effective code diffusion strategy which can reduce delay and transmission times simultaneously. The main innovations of this paper are as follows:

(1) We first proposed a code diffusion scheme called If Fail Add Slot (IFAS). In the IFAS scheme, parent node sends the data successively according to the awake slots of its son nodes, so the son nodes with small serial numbers of awake slot receive the data first. Unlike previous strategies, in IFAS scheme, when the son nodes with small serial number of awake slot fail to receive data, they will be re-awake at the next node’s awake slot. In this way, when parent node sends data to the next son node, the former nodes can also try receiving data. If the node fails again, it will continue to wake up when parent node sends data until the reception is successful. Obviously, IFAS can effectively reduce delay and transmission times. Because, in previous strategies, as long as the son node does not receive successfully, the parent node will send again at the awake slot of this node in the next cycle, so the additional delay is *T* and the additional sending times is 1. In IFAS, there is almost no increase in transmission times, because when these nodes fail to receive data successfully, they will receive data again when parent node sends data to other son nodes, which makes full use of the broadcasting feature of wireless channel and does not increase the times of sending. Meanwhile, in IFAS scheme, node can receive data several times in one cycle, so it is possible to receive data successfully in one cycle instead of several cycles even if receiving fails multiple times, which greatly reduces delay and transmission times. Obviously, by reducing transmission times, energy consumption is also reduced, which means lifetime is prolonged. Therefore, IFAS makes breakthrough where previous strategies could only accomplish tradeoff between multiple performances and achieves optimization of multiple performances at the same time, which is the first innovation point of this paper

(2) A Before Try Add Slot (BTAS) scheme is proposed that can further improve performance. While IFAS allows packets that need resending to be received within a cycle without increasing the number of deliveries, which is beneficial to the son nodes with small slot serial number, because it can add awake slots when the reception fails, thus reducing the delay and transmission times without increasing the energy consumption, it is not very efficient to the node with the largest slot serial number. Because this son node is the last node to receive data from its parent node. If the sending is unsuccessful, it can add awake slot to reduce delay and transmission times when parent node sends data to the first node in the next cycle. However, this means the transmission to this node needs two cycle *T* to be completed. Although it is still better than previous strategies even in such a bad situation, we have found that the performance of IFAS can be further bettered. We have discovered that when nodes collect data, the nodes near sink take up a large amount of data and consumes a large amount of energy, while the nodes away from sink have surplus energy. Therefore, BTAS allots some awake slots which belong to the brother nodes that wake up earlier in a cycle to the son node with the largest slot serial number in area away from sink. In this way, BTAS can improve the performance of all son nodes by the same margin.

(3) An Add Average Place Slot (AAPS) scheme is proposed to further improve networks performance. Because in the strategies proposed previously, compared with traditional strategy, the number of awake slots added is fairly small. Therefore, there is still energy left over in area away from sink node, which can be exploited to add awake slots in the beginning of working cycle, thus allowing code to be propagated timely, so that delay can be further reduced. AAPS can also simultaneously reduce delay and transmission times and prolong lifetime, but, in contrast with previous strategies, it may consume a bit more transmission times, yet, since the transmission times is increased in area with energy surplus, the effect on lifetime is little. 

(4) The strategies proposed in this paper make breakthrough where previous strategies cannot achieve all-around optimization, which means they can optimize delay, transmission times and lifetime at the same time, which is a huge step forward. After a lot of theoretical and experimental analysis, compared to the traditional method, IFAS, BTAS, AAPS scheme can respectively reduce delay by 20.56%, 31.59%, 55.16% and transmission times by 29.53%, 43.93%, 42.04%, while not reducing lifetime.

The rest of this paper is organized as follows: In Section 2, the related work is stated. Then, the networks model and problem statement is introduced in Section 3. IFAS, BTAS, AAPS schemes are respectively illustrated in Section 4. Theoretical performance analyses of proposed schemes are presented in Section 5. Results of simulation and analysis of experimental performances are presented in Section 6. Finally, Section 7 provides conclusions.

## 2. Related Work

WSNs consists of a huge number of devices composed of microprocessor, power supply equipment, memory and communication equipment [52,53,54] and is an important component of Internet of Things (IoT). With the development of microprocessors, the processing and perceiving ability of sensor nodes is getting stronger and stronger, greatly expanding the scope and field of application, therefore greatly promoting the development of IoT [55,56]. Since these sensor nodes already have the same processing power and function as, or even stronger than, that of a PC produced 10 years ago and the amount of data processed by these sensor nodes or devices equipped with sensor nodes is extremely large, the development of networks is profoundly affected. According to scientific research [2], since 2011, the number of devices connected to Internet of Things (smartphones, city-monitors and industrial-awareness devices, etc.) on earth has exceeded population, reaching 9 billion. It is estimated that by 2020, 24 billion devices will be connected to networks [2]. This situation has promoted the emergence of new computing models, such as edge computing, big data networks and so forth. These new computing models combined with cloud computing have brought new opportunities and challenges to networks [57,58].

Another technology that has promoted the development of networks is SDNs [7,13,15]. SDNs is a technology that allows hardware to work like software. In such technology, the hardware of networks equipment performs the basic functions, while it is software-based, that is, the function of networks equipment can be redefined by changing the software of it through solid-state software, thus making it more flexible and able to update the software at any time to customize the networks adaptively [13,15].

The above technologies bring both opportunities and new challenges to WSNs. Among them, the update and the soft code diffusion of WSNs are summarized as the following most important types of research based on different targets of optimization.

### 2.1. Research on Delay Optimization

There have been some research on code diffusion and one of the most important goals of code diffusion in WSNs optimization is delay optimization. Obviously, delay should be as small as possible. These research can be divided into two types, one of which is directed at duty cycle based WSNs [7,13,15]. In such networks, every node adopts the approach of periodical sleep/awake independently. For the WSNs with low duty cycle, there is only one awake slot during a working cycle, while the node is asleep in other slots, which is much more energy-efficient. In WSNs that have bigger duty cycle, nodes are allowed to have more than one awake slots in a cycle, which apparently makes it easier for the code to be propagated, while it calls for more energy. Following is a study that is most relevant to this paper [51].

**Figure 1:**
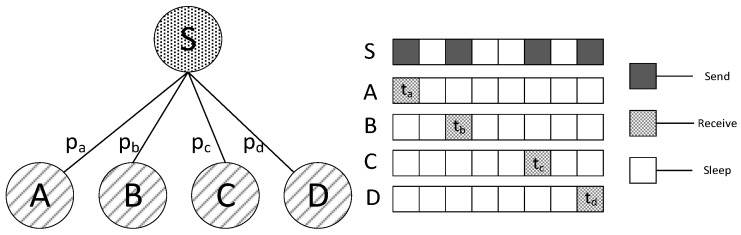
Traditional code diffusion scheme.

In this type of scheme, code dissemination can be illustrated by Figure 1. We assume that the sink node S is responsible for sending the code to its four son nodes, namely node A, B, C, D and their awake slots are$\;t_{a},\;t_{b},\;t_{c},\;t_{d}$. Thus, the sink node performs the sending operation respectively at slot $t_{a},\;t_{b},\;t_{c},\;t_{d}$ (see Figure 1). However, due to the unreliability of wireless communication, the percentage of successful transmission from sink to each son node is $\;p_{a},\;p_{b},\;p_{c},\;p_{d}$. Therefore, after one round of transmission to each son node, there are possibly some nodes that have not got the code successfully. Then, to those son nodes without code, sink will send the code again during the next working cycle at corresponding slot; should there still be nodes yet without code, code will be sent again in next cycle. In Ref. [51], the expected transmission times is 1/p, where p denotes the successful rate of one-time transmission. According to the scheme above, Ref. [51] gives the code diffusion method of the entire networks. The essence of it is a tradeoff between delay and transmission times. First, the networks are abstracted to a tree with sink as root. For every two nodes that are able to communicate, they are connected by an edge, the weight of which is 1/p, the expected transmission times. Trees built in this way, when minimum spanning tree algorithm is used, will achieve smallest expected transmission times. However, the delay in this case may not be the smallest. The shortest path scheme based on the shortest path from sink to each node is able to achieve the smallest delay, while the transmission times may not be optimal. Therefore, Ref. [51] put forward a tradeoff scheme between delay and transmission times. The essence of it is a combined scheme of minimum spanning tree and shortest path. However, one of the deficiencies is that delay is still quite huge. In fact, even when shortest path is realized, the delay is still huge. The ultimate cause is that when code is not received successfully, delay will be increased by a cycle. And, when the delay of previous nodes increases, that of descendant will increase as a result. Therefore, delay of the whole networks also increases. Actually, the transmission times of this scheme is big as well, since the broadcasting nature of wireless communication, is not fully utilized, which also has effects on energy consumption and lifetime under this scheme. While in the method proposed by us, since we allow the node to wake up as quickly as possible instead of waking up in the next cycle, delay is largely reduced. And, because we utilize the broadcasting nature, there are possibly several nodes sharing the same slot so that the transmission times is also reduced.

There are still other methods concerning code dissemination in WSNs, one of them being gossiping [47]. Gossiping is widely used in networks where it is not obliged to ensure every node receives data with small delay. Since it has very low environmental requirements and transmission costs, its application is versatile in delay-tolerating networks. However, in this paper, the networks we study is different: It is not delay-tolerating. It has to ensure every node receives data. The code transmission is conducted in prescriptive slots along decided route instead of random gossiping. Therefore, the method we propose is much more suitable.

### 2.2. Research on Transmission Times

In such type of research, the main target is to reduce the times of node’s broadcasting/transmitting, namely to deal with the minimum-transmission broadcast (MTB) issue. This research can also be divided into two types: duty cycle based WSNs and non-duty cycle based WSNs. In non-duty cycle based WSNs, since the node is always active, the code can be disseminated at any time as long as there is no interference in the channel. However, if every node is broadcasting, it will cause broadcast storm, thus greatly consuming energy. Therefore, in order to reduce transmission times, the approach adopted in Ref. [59,60] is to find a Minimum Connected Dominating Set (MCDS) of the networks. MCDS is a set of nodes with the characteristics like these: there is a route connecting any two nodes in this set and in the meantime, any node of this networks is a neighbor one-hop away from a node in the set, which means if all the nodes in the set broadcast once, any node in the networks can receive the code. Apparently, if the code is sent to every node in MCDS, the code dissemination can be completed in the entire networks with one transmission from each node of MCDS. Obviously, the number of nodes in MCDS is much smaller than that of the whole networks, therefore the transmission times can be greatly reduced. A similar work is minimum flooding tree designed in Ref. [61] and it is proved by the author that this method is equal to MCDS.

But, this method needs adapting before applied to duty cycle based WSNs. Since in duty cycle based networks, nodes rotate between sleep and awake modes periodically, only one transmission is far from enough to allow all nodes to receive code. When sender is broadcasting, a certain number of nodes may be in sleep mode, thus unable to receive code. As a result, the MTB problem in duty-cycled networks (MTB-DC problem) gets more complex and challenging. Similar to MCDS, MTB-DCL problem can also be addresses with a broadcast backbone. Then, after the slots and broadcasting time are carefully planned, the code can be first disseminated to every node in the broadcast backbone quickly, from which, after one or several transmissions, code can be sent to every node in the networks. Duc and his colleagues in Ref. [11] proposed a Level-Based Approximation Scheme based on the thought described above. In their scheme, it is assumed that nodes in broadcast backbone do not rotate between sleep/awake, instead, they are always active to build up broadcast backbone (similar to MCDS) and then code is disseminated into the whole networks through broadcast backbone. Research aimed at MTB-DC problem also include: Zhao D in Ref. [7] presented two approximation algorithms, BS-1 and BS-2. Khiati M in Ref. [62] proposed a Broadcast over Duty-Cycle and LEACH (BOD-LEACH) protocol.

In fact, the delay in MTB-DC problem is mainly decided by the time needed to build broadcast backbone. Because, after broadcast backbone is built, it only requires at most a constant time |T| to finish the dissemination in the whole networks. And duty cycle is an important element when building broadcast backbone. If a node can have several active slots in a cycle  T, apparently, it is possible to conduct code transmission for several times in a cycle, therefore the time needed to build broadcast backbone is shorter, thus reducing transmission time. Apparently, if the active slots of a node in a cycle can be added, the possibility of sending data is increased and the possibility of several nodes’ receiving data at a same slot during broadcast is also increased, thus accelerating the dissemination of code. However, when adding active slots, nodes consume more energy. So lifetime may be affected. But, we have discovered that in WSNs, when conducting data collection, nodes near sink node take up more data and consume more energy while nodes away from sink take up less data and consume less energy [63]. Therefore, if the energy surplus is fully utilized, delay can be reduced while lifetime is not affected. Based on the thought described above, we proposed an adjustable duty cycle based fast disseminate (ADCFD) scheme that can effectively reduce code diffusion delay [6]. A few other research dealing with energy-efficiency are in Ref. [64,65].

In addition, the speed of code dissemination is also related to the radius of broadcast. Apparently, the longer is the radius, the larger is the number of nodes that are able to receive code and the further is the distance of one transmission. In this way, code can be disseminated faster in the whole networks with less transmission times. However, since it will increase energy consumption to broadcast with longer radius, the lifetime may well be affected. In Ref. [15], we proposed a code dissemination scheme based on unequal radius. The basic thought of this scheme is that the sending radius in area away from sink with energy surplus is enlarged while it remains the same in area near sink. After experiment and theoretical analysis, it is demonstrated that the proposed scheme can effectively reduce delay while not affecting lifetime [15].

### 2.3. Research on Reliability

In previous research, it is assumed that wireless communication channel is ideal, without packets loss, therefore only one transmission is required to complete code dissemination. However, in reality the packet loss ratio of wireless communication is way higher than that of wired networks. When the surrounding environment is complex, the loss ratio is even higher. According to relevant research [50]: the packet loss ratio of wireless networks can be over 30%. Therefore, the reliability of transmission needs considering in wireless communication. Retransmission scheme is one of the most widely used and effective ways to solve the problem of unreliable transmission, that is, when sender fails to send the data, it will resend the data again. Thus, in consideration of retransmission, some of the schemes mentioned above is no longer suitable. The current scheme is demonstrated in Figure 1, which adopts the method of retransmission at the awake slot in the next cycle when transmission fails. Obviously, we have already explained why this method is not effective. And, as stated before, by allowing the node to wake up as quickly as possible in the same cycle, our method can sufficiently reduce the delay.

This paper is focused on WSNs with packet loss and low duty cycle. Therefore, the complexity and difficulty of this research has surpassed previous ones, especially when trying to reduce delay and transmission times at the same time without shortening lifetime.

## 3. Networks Model and Problem Statement

### 3.1. Networks Model

**Figure 2:**
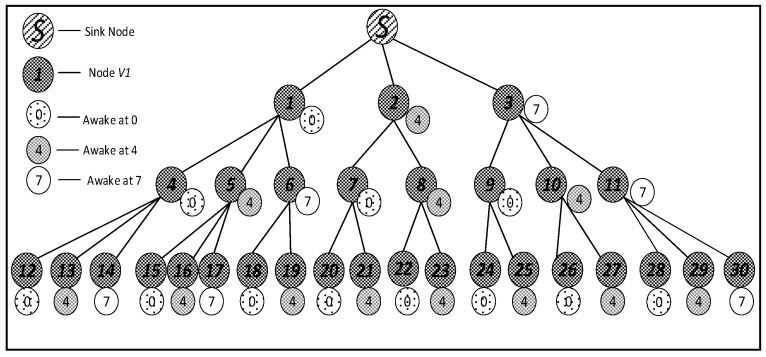
Networks topology.

The networks model adopted in this paper is the same as that of Ref. [53]. The nodes are evenly distributed in a circular region with radius R, with a root node $S_{base}$ and n identical source nodes $V=\left\{v_{1},v_{2},\ldots \ldots v_{n}\right\}$. Thus, the entire networks can be represented as $W=\{S_{base},v_{1},v_{2},\ldots \ldots v_{n}\}$, where $v_{i}$ is the node ID. Sink node is located at the center of the circle. Node density is ρ, according to definition, $\rho =\frac{n}{\pi R^{2}}$. Similar networks with that in Ref. [53] can be converted into a tree with sink node as the root of the tree, as shown in Figure 2 in Section 4.1. The main functions of sensor networks are data sensing and the transmission of perceived data through multiple hops routed to sink. The data collected by each node is first sent to its parent node and then passed through the parent nodes step by step, until it reaches$\;S_{base}$.

Source nodes are driven by the same battery and have limited energy. The energy of source nodes is set as $E_{battery}$ and the root node has unlimited energy. Each parent has a different number of son nodes. In order to save energy, all nodes have two modes: awake and sleep. During one working cycle, nodes wake up only at one slot and sleep at the others. A working cycle is divided into m time slots shared by all nodes, {$s_{1},s_{2},\ldots \ldots ,s_{m}$}. During initialization, each node randomly selects its own awake slot and informs its parent. The awake slots of the brother nodes can be obtained by asking each other. The successful rate of single-time transmission is$\;P_{trans}$. To prevent multiple retransmission of the same data packet, the maximum transmission times is set to $T_{max}$ according to$\;P_{trans}$, in which $T_{max}$ is the minimum transmission times for the total success ratio to reach a threshold$\;P_{th}$.

Code diffusion is the main research content of this paper. The sensor networks belong to SDNs, which means networks functions can be updated by spreading code irregularly. During code diffusion, starting from the root node$\;S_{base}$, packets are delivered from parent to son nodes and then, if son nodes receive the packet correctly, they will send it to their own son nodes, to realize data packet broadcast. The parent node knows when the son nodes wake up and sends the packet to them at corresponding slots. When the parent node confirms that the packet is received correctly, it stops sending the packet.

There are three types of energy consumption that exist in the model when broadcasting. The first type is the energy consumed when the parent node sends the packet to the son node (denoted as$\;E_{trans}$); the second type is the energy consumed when the son node receives the packet from the parent node (denoted as$\;E_{receive}$); the third type is the energy consumed when the son node wakes up to detect whether the parent node sends the packet to it or not (denoted as$\;E_{awake}$). It is assumed that $E_{awake}$ is included in$\;E_{receive}$. And it is assumed that the packet sent is of the same size, so the energy used to send and receive the packet is unchanged, in other words, $E_{trans}$ and $E_{receive}$ maintain the same during the whole process.

### 3.2. Problem Statement

**Definition 1.**  *Minimum broadcast delay (denote broadcast delay as* D*). In this paper,*$Delay\left(v_{i}\right)$*of node*$v_{i}$*is defined as the number of slots between sending data packets from*$S_{base}$*and successful reception of data packets by*$\;v_{i}$*. General broadcast delay of the networks is*$\;\sum_{v_{i}\in V}Delay\left(v_{i}\right)$. *Therefore, the goal of this paper in the aspect of delay can be summarized as the following formula:*

$$\;\min\left(D\right)=\min\left(\sum_{v_{i}\in V}\mathrm{Delay}\left(v_{i}\right)\right).$$

**Definition 2.**  *Minimum transmission times (denote transmission times as* T*). In this paper, the*$\;TransmissionTimes\left(v_{i}\right)$*of the parent node is defined as the number of times that the parent node*$\;v_{i\;}$*sends the packet to all of its sons until they successfully receive the packet. General transmission times of the networks is*$\;\sum_{v_{i}\in V}TransmissionTimes\left(v_{i}\right)$. *Therefore, the object of this paper in terms of transmission times can be summarized as the following formula:*

$$\min\left(T\right)=\min\left(\sum_{v_{i}\in V}\mathrm{TransmissionTimes}\left(v_{i}\right)\right).$$

**Definition 3.**  *Minimum energy consumption (denote energy consumption as* E*). In this paper,*$EnergyConsumption\left(v_{i}\right)$*of node*$v_{i}$*is defined as the energy which*$v_{i}$*uses to receive the packet, send the packet and wake up during the whole time from when*$\;S_{base}$*sends the data packet to when all the nodes receive the data packet.*$\;EnergyConsumption\left(v_{i}\right)=aE_{trans}+bE_{receive}+cE_{awake}$*. a, b and c are respectively the number of times*$v_{i}$*sends the packet, the number of times*$v_{i}$*receives the packet and the number of times*$v_{i}$*wakes up during the code dissemination in the whole networks. General energy consumption is thus*$\;\sum_{i\subseteq V}EnergyConsumption\left(v_{i}\right)$. *Therefore, the goal of this paper in energy consumption can be summarized as the following formula:*

$$\min\left(E\right)=\min\left(\sum_{v_{i}\in V}\mathrm{EnergyConsumption}\left(v_{i}\right)\right).$$

Therefore, the problems dealt with in this paper can be expressed by the following formula:

$$\{\begin{array}{c} \min\left(D\right)=\min\left(\sum_{v_{i}\in V}\mathrm{Delay}\left(v_{i}\right)\right)\\ \min\left(T\right)=\min\left(\sum_{v_{i}\in V}\mathrm{TransmissionTimes}\left(v_{i}\right)\right)\\ \min\left(E\right)=\min\left(\sum_{v_{i}\in V}\mathrm{EnergyConsumption}\left(v_{i}\right)\right) \end{array}.$$

For the convenience of readers, Table 1 summarizes the symbols used in the paper, their meanings and their values.

## 4. Minimum Delay Scheme Design

### 4.1. Research Motivation

In this section, a specific network is used to compare the different performances to state the research motivation. The networks topology adopted is shown in Figure 2. In such networks, the working cycle T = 8, which means a cycle consists of eight slots, numbered {0, 1, 2, 3, 4, 5, 6, 7}. Suppose all the first son nodes of all the parent nodes in the figure wake up at slot 0 and the maximum retransmission number is set to 3. The second son nodes wake up at slot 4 and the maximum number of retransmission is set to 2. The third son nodes (if any) wake up at slot 7 and the maximum number of retransmission is set to 3.

**Figure 3:**
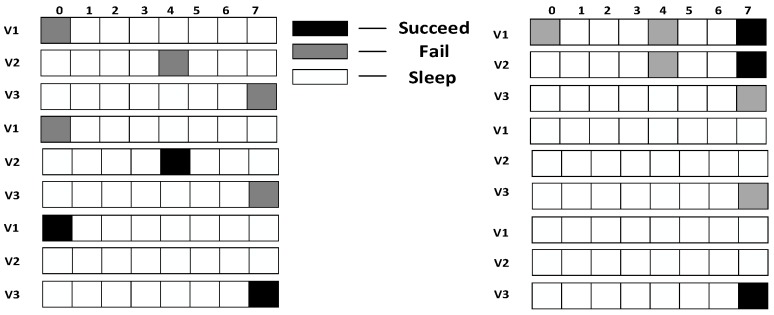
Traditional diffusion scheme (**left**) and IFAS (**right**).

Research motivation is illustrated by describing how the sink node in Figure 2 transfers code to $v_{1},\;v_{2},\;v_{3}$. The scheme in comparison is that proposed in Ref. [53]: parent node sends code at each of its son nodes’ wake slot. If son node fails to receive code, parent node will resend at the same slot in the next cycle. Therefore, the diffusion process of code to $v_{1},\;v_{2},\;v_{3}$ using traditional scheme is illustrated in Figure 3 (left). Every node only receives packet at its own slot. $v_{1}$ fails during the first and the second cycle and succeeds in the third cycle at its own slot, $\mathrm{Delay}\left(v_{1}\right)=16;v_{2}$ fails during the first cycle and succeed at its own slot in the second cycle, $\mathrm{Delay}\left(v_{2}\right)=12;v_{3}$ fails during the first and the second cycle and succeeds in the third cycle at its own slot, $\mathrm{Delay}\left(v_{3}\right)=23$.

According to If Fail Add Slot (IFAS) scheme, after parent node has sent code to its son node, if the reception fails, the node will wake up again next time when parent node sends code to its brother node and if it fails again, the node will wake up again when parent node sends code to other nodes. Since the node is destined to wake up again to receive code if the reception fails, IFAS does not increase the times of reception, therefore not increasing energy consumption. And to the sender (parent node), instead of adding sending times, the broadcast nature of radio is used to allow several nodes to receive data simultaneously. Using this approach, it avoids the sending operation in the next cycle that is destined in traditional scheme, meanwhile, delay is also reduced. The sending process is illustrated in Figure 3 (right). In the first cycle, $v_{1}\;$fails at its own slot and wakes up again at$\;v_{2}$’s slot. This time, $v_{1}$ also fails. Then, it wakes up again and succeeds at $v_{3}$’s slot, $\mathrm{Delay}\left(v_{1}\right)=7;v_{2}$ fails to receive code at its own slot and then it wakes up again at $v_{3}$’s slot and succeds in receiving, $\mathrm{Delay}\left(v_{2}\right)=7;v_{3}$ fails at its own slot in the first cycle and in the second cycle it fails as well, finally it succeeds in the third cycle at its own slot, $\mathrm{Delay}\left(v_{3}\right)=23$.

Table 2 shows the delay of all the nodes in the model shown in Figure 2 when the traditional diffusion method is adopted and the average delay is 45.9. The total number of deliveries is 78, with an average of 2.6.

Table 3 shows the delay of all the nodes in the model shown in Figure 2 when IFAS is adopted and the average delay is 32.47. The total number of deliveries is 54 and the average is 1.8. Compared with traditional scheme, the average delay is decreased by 29.26% and the average transmission times is decreased by 30.77%.

**Figure 4:**
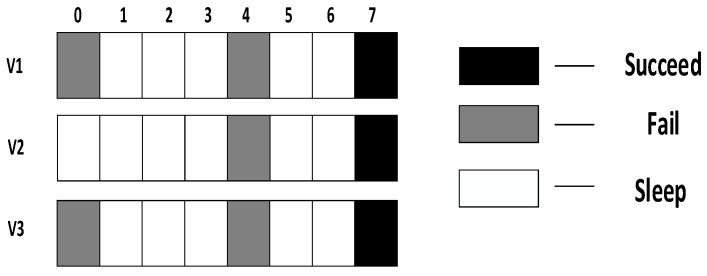
BTAS method.

In the IFAS mode, since $v_{1}$ wakes up first in the working cycle, if it fails to receive at its own slot, there will be two more chances. $v_{2}\;$wakes up second. If it fails to receive at its own slot, there will be one more chance. Therefore, IFAS method significantly reduces $\mathrm{Delay}\left(v_{1}\right)$ and D$\mathrm{elay}\left(v_{2}\right)$. However, since $v_{3}$ is the last node to wake up, the IFAS method cannot be used to improve performance. Therefore, on the basis of IFAS, we proposed the Before Try Add Slot (BTAS) strategy. Since the nodes away from sink have energy surplus, for the last waking nodes like$\;v_{3}$, this strategy adds awake slots at the slots of other son nodes who wake up earlier, thereby reducing delay. Its specific sending process is shown in Figure 4, while the process of $v_{1}$ and $v_{2}$ is the same as that of IFAS, $v_{3}$ adds an active slot at the slot of $v_{1}$, at which the receiving fails, then it wakes up at the slot of $v_{2}$, at which the receiving fails again and finally it succeeds at its own slot,$\;\mathrm{Delay}\left(v_{3}\right)=7$.

Table 4 shows the delay of all the nodes in the model shown in Figure 2 when using the BTAS method and the average delay is 22.87. The total number of deliveries is 42, with an average of 1.4. In comparison with the traditional scheme, the average delay is decreased by 57.95%, the average transmission times is decreased by 46.15%. In comparison with IFAS, the average delay is decreased by 29.57% and the average transmission times is decreased by 22.22%.

In the traditional method, since the son node wakes up at only one slot in a cycle, while there are many slots in one cycle, when the parent node is ready for the code to be sent, it often needs to wait a long time until the son node wakes up, so it can send code to the son node, thus causing huge delay. The nodes away from the sink have energy surplus, therefore, they can have extra awake slots to receive code. The extra slots added are decided in this way: they are located at the place where the active slots are most evenly distributed in the working cycle, that is, the location which makes the expected delay the least. After the slots are added, the node will continue to work in IFAS method. This reduces the delay of the parent’s waiting for son nodes to wake up, which is unavoidable in the traditional method. At the same time, since the son nodes of the same parent node all add slots, the coverage of slots in a cycle is improved, so that when the node fails to receive, the time it waits for the next slot when code is available is reduced, further improving the performance of the IFAS algorithm. Therefore, using this method can reduce both the delay of waiting for the son node to wake up and the delay of waiting for the next slot after failure.

**Figure 5:**
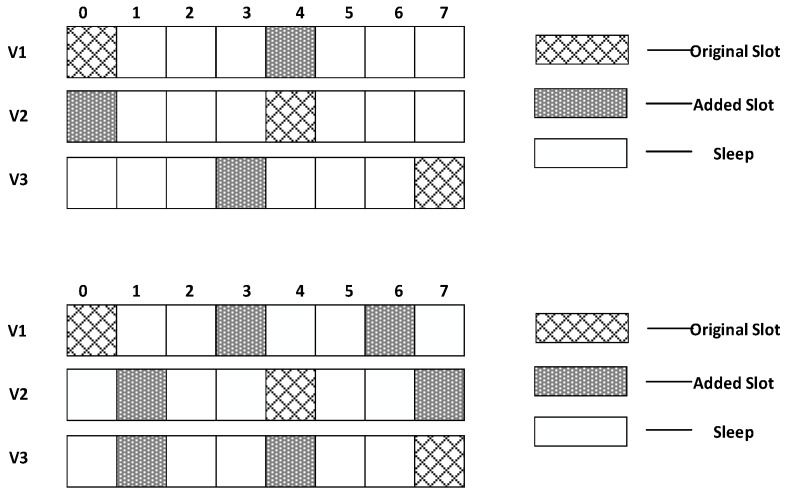
The added slots in the third layer (**top**) and the added slots in the fourth layer (**below**).

In the model shown in Figure 2, we assume that layer 3 has enough energy to add one slot and layer 4 has enough energy to add two slots. Therefore, as shown in Figure 5 (top), in the third layer, the nodes that wake up at slot 0 add one slot at slot 4; the nodes that wake up at slot 4 add one slot at time 0; the nodes that wake up at slot 7 add one slot at slot 3. In the fourth layer, as shown in Figure 5 (below), the nodes that wake up at slot 0 add two slots at slot 3 and slot 6; the nodes that wake up at slot 4 add two slots at slot 1 and slot 7; the nodes that wake up at slot 7 add two slots at slot 1 and slot 4.

Table 5 shows the delay of all the nodes in the model shown in Figure 2 when AAPS is adopted and the average delay is 21.43. The total number of deliveries is 44, with an average of 1.47. Compared with the traditional scheme, the average delay is reduced by 53.31% and the average transmission times is reduced by 43.46%. Compared with IFAS, the average delay is reduced by 34.00% and the average transmission times is reduced by 18.33%. Compared with BTAS, the average delay is reduced by 6.30% and the average transmission times is increased by 5%.

Since the node $v_{3}$ in Figure 2 adds a slot when there is not enough energy to add awake slots in this method, the delay of $v_{3}$ and its descendant nodes is greatly reduced. However, even in this case, AAPS method can still further reduce the delay than BTAS method. Since the AAPS method increases transmission times in the nodes away from sink, thus reducing the delay, the average transmission times is slightly more than that of BTAS, still less than that of traditional scheme and IFAS.

### 4.2. IFAS, BTAS, AAPS Schemes Design

In the traditional method, because the slots of the son nodes are not uniformly distributed, the parent node can only send packets to son nodes individually when they wake up. The algorithm is described below:

Suppose a parent node has z son nodes and they have randomly generated working slots,$\;\{s_{1},s_{2},\ldots \ldots ,s_{z}\}$.

Assuming that the parent node finishes receiving data packets at slot$\;s_{j}$, from the slot$\;s_{j+1}$, if there is any slot, at which there is a son node waking up, the parent node will broadcast the data. Then the record of transmission times to this son node will add 1 (initialized to 0). The parent node will not send the packet at this slot again when either of the following two conditions is met:

1. The transmission times to this son node reaches $T_{max}$
2. Receive ACK from the son node

One notable drawback of traditional method is that each son node has only one chance to receive packets within a cycle and if it fails, it has to wait one cycle to try again. After observing this, we improve it and use the IFAS method to let the son nodes inform each other of the awake slots of their own. When the son node fails to receive the packet, it can wake up and try to receive the packet again at brother nodes’ slots. The description of the algorithm is as follows:

Suppose a parent node has z son nodes and they have randomly generated working slots,$\;\{s_{1},s_{2},\ldots \ldots ,s_{z}\}$.

The son nodes ask each other and record the slots later than their own. For all z nodes, the slots they recorded are:

{$s_{1}$,$s_{2}$,……$s_{z}$},

{$s_{2}$,$s_{3}$,……$s_{z}$},

{$s_{3}$,$s_{4}$,……$s_{z}$},

......

{$s_{z-1},s_{z}$},

{$s_{z}$}

Assuming that the parent node finishes receiving data packets at slot$\;s_{j}$, from the slot$\;s_{j+1}$, if there is any slot, at which there is a son node waking up, the parent node will broadcast the data. Then the record of transmission times to this son node will add 1 (initialized to 0). The parent node will not send the packet at this slot again when either of the following two conditions is met:

1. The transmission times to this son node reaches $T_{max}$
2. Receive ACK from all the son nodes

For the son node$\;v_{i}$, the slot list is {$s_{i}$,……$s_{z}$}. From the slot$\;s_{i}$, $v_{i}\;$begins to receive the packet and if the receiving fails, then it will wake up at the next slot in the slot list and try to receive the packet. The son nodes will stop waking up at other slots when either of the following two conditions is met:

1. The packet is received successfully
2. It senses nothing at its own slot$\;s_{i}$

Initializing process of father node under IFAS scheme can be described by Algorithm 1.

**Algorithm 1** Initialize father node under IFAS scheme1: Son_Count = 0 2: **For** each node of Son_Nodes
**Do** 3:  Add this node’s awake slot to father node’s sending slot list 4:  Son_Count = Son_Count + 1 5: **End for**

Initializing process of son nodes under IFAS scheme can be described by Algorithm 2.

**Algorithm 2** Initialize son nodes under IFAS scheme1: **For** each node of Brother_Nodes **Do** 2:  **If** this brother node’s awake slot > own awake slot **then** 3:  add this brother node’s awake slot to receiving slot list 4:  **End if** 5: **End for**

Working procedure of father node under IFAS scheme can be described by Algorithm 3.

**Algorithm 3** Broadcast data packet under IFAS scheme1: Success_Count = 0 2: **While** Success_Count < Son_Count **Do** 3:  wait until the next slot in sending slot list 4:  broadcast data packet 5:  **For** each node of Son_Nodes
**Do** 6:    **If** this node receives successfully **then** 7:    Success_Count= Success_Count + 1 8:    **End if** 9:  **End for** 10: **End while**

Working procedure of son nodes under IFAS scheme can be described by Algorithm 4.

**Algorithm 4** Receive data packet under IFAS scheme1: Success_Flag = 0, Receive_Flag = 0 2: **While** Receive_Flag==0 **Do** 3:  wait until own slot 4:  **If** this node receives successfully **then**
5:   Success_Flag = 1, Receive_Flag = 1 6:  **End if** 7:  **Else if** this node receives unsuccessfully **then** 8:   Success_Flag = 0, Receive_Flag = 1 9:  **End else** 10: **End while** 11: **While** Success_Flag==0 **Do** 12:  wait until the next slot in receiving slot list 13:  **If** this node receives successfully **then** 14:   Success_Flag = 1 15:  **End if** 16: **End while**

The IFAS method effectively makes use of the feature that the parent node sends the data packets multiple times in a cycle, so that a son node can try to receive the data packets multiple times in a cycle. One problem is that the last waking node cannot attempt to receive packets at other slots, so based on IFAS, we design the BTAS method to add slots in the front of the cycle for nodes that wake up the last. The description of the algorithm is as follows:

Suppose parent node has z son nodes and they have randomly generated working slots,$\;\{s_{1},s_{2},\ldots \ldots ,s_{z}\}$.

The son nodes ask each other and record the slots later than their own. For all z nodes, the slots they recorded are:

{$s_{1}$,$s_{2}$,……$s_{z}$},

{$s_{2}$,$s_{3}$,……$s_{z}$},

{$s_{3}$,$s_{4}$,……$s_{z}$},

......

{$s_{z-1},s_{z}$},

{$s_{z}$}

For nodes with only one slot in the slot list, they will add a wake slot at the slot $s_{1}$ at which there is a node waking up in the cycle for the first time and then tell the parent to send data to them at $s_{1}$. Then the intermediate slots are also added. Therefore, the updated slot lists are:

{$s_{1}$,$s_{2}$,……$s_{z}$},

{$s_{2}$,$s_{3}$,……$s_{z}$},

{$s_{3}$,$s_{4}$,……$s_{z}$},

......

{$s_{z-1},s_{z}$},

{$s_{1}$,$s_{2}$,……$s_{z}$}

Assuming that the parent node finishes receiving data packets at slot$\;s_{j}$, from the slot$\;s_{j+1}$, if there is any slot, at which there is a son node waking up, the parent node will broadcast the data. Then the record of transmission times to this son node will add 1 (initialized to 0). The parent node will not send the packet at this slot again when either of the following two conditions is met:

1. The transmission times to this son node reaches $T_{max}$
2. Receive ACK from all the son nodes

For the son node $v_{i}$, the slot list is {$s_{i}$,……$s_{z}$}. From the slot$\;s_{i}$, $v_{i}\;$receives the packet and if the reception fails, then it will wake up at the next slot in the slot list and try to receive the packet. The son nodes will stop waking up at other slots when either of the following two conditions is met:

1. The packet is received successfully
2. It senses nothing at its own slot$\;s_{i}$

Initializing process of father node under BTAS scheme can be described by Algorithm 5.

**Algorithm 5** Initialize father node under BTAS scheme1: Son_Count = 0 2: **For** each node of Son_Nodes
**Do**
3:  Add this node’s awake slot to father node’s sending slot list 4:  Son_Count = Son_Count + 1 5: **End for**


Initializing process of son nodes under BTAS scheme can be described by Algorithm 6.

**Algorithm 6** Initialize son nodes under BTAS scheme1: After_Slots_Count = 0,Active_Slot = m 2: **For** each node of Brother_Nodes **Do** 3:  **If** this brother node’s awake slot > own awake slot **then** 4:  add this brother node’s awake slot to receiving slot list 5:  After_Slots_Count = After_Slots_Count + 1 6:  **End if** 7: **End for** 8:**If** After_Slots_Count==0 **then** 9: **For** each node of Brother_Nodes **Do** 10:   **If** this brother node’s awake slot < Active_Slot **then** 11:   Active_Slot = this brother node’s awake slot 12:   **End if** 13: **End for** 14: Add Active_Slot to awake slot list 15:**End if** 16:Add own slot to awake slot list

Working procedure of father node under BTAS scheme can be described by Algorithm 7.

**Algorithm 7** Broadcast data packet under BTAS scheme1:Success_Count = 0 2:**While** Success_Count < Son_Count **Do** 3:  wait until the next slot in sending slot list 4:  broadcast data packet 5:  **For** each node of Son_Nodes
**Do** 6:   **If** this node receives successfully **then** 7:   Success_Count = Success_Count + 1 8:   **End if** 9:  **End for** 10: **End while**

Working procedure of son nodes under BTAS scheme can be described by Algorithm 8.

**Algorithm 8** Receive data packet under BTAS scheme1: Success_Flag = 0,Receive_Flag = 0 2:**While** Receive_Flag==0 **Do** 3:  wait until next slot in awake slot list 4:  **If** this node receives successfully **then**
5:   Success_Flag = 1, Receive_Flag = 1 6:  **End if** 7:  **Else if** this node receives unsuccessfully **then** 8:   Success_Flag = 0, Receive_Flag = 1 9:  **End else** 10:**End while** 11: **While** Success_Flag==0 **Do** 12: wait until the next slot in receiving slot list 13: **If** this node receives successfully **then** 14:   Success_Flag = 1 15: **End if** 16:**End while**


Since the nodes away from the sink node forward less data and have a lot of energy left over, we can add more slots to listen for packets from the parent node to minimize latency. To sum up the IFAS and BTAS methods, it can be concluded that the earlier the son node knows that the parent starts to transmit the data packet, the more slots of brother nodes the son node can use to receive data packets after failure. In addition, as the number of slots with son nodes waking up increases, the parent node will send packets more frequently and reduces latency when multiple transmission is required. AAPS algorithm is designed according to the above two points. The description of the algorithm is as follows:

Suppose a parent node has z son nodes and they have randomly generated working slots,$\;\{s_{1},s_{2},\ldots \ldots ,s_{z}\}$.

First, the son node calculates the number of slot (denoted as d) that can be added based on its own data volume and then determines the location of the added slot in this way:

1. Calculate the number of sleeping slots, w=m−d−1
2. Update w to average number of sleeping slots, w=w/(d−1)
3. Starting from the slot randomly generated by the son node itself, it first adds a slot with a gap of ⎡w⎤−1, then adds another slot with a gap of ⎡w⎤ and so on, until the number of added slot reaches d. If the node has reached the end of the cycle, then it will continue adding slots from the beginning of the cycle.

After the son nodes have added slots, they will send their full slot list to the parent node.

The son nodes ask each other and record all the brothers’ slots. Thus, supposing that they have e different awake slots, the slots stored by all nodes are$\;\{s_{1},s_{2},\ldots \ldots ,s_{e}$}.

Assuming that the parent node finishes receiving data packets at slot$\;s_{j}$, from the slot$\;s_{j+1}$, if there is any slot, at which there is a son node waking up, the parent node will broadcast the data. Then the record of transmission times to this son node will add 1 (initialized to 0). The parent node will not send the packet at this slot again when either of the following two conditions is met:

1. The transmission times to this son node reaches $T_{max}$
2. Receive ACK from all the son nodes

For son node$\;v_{i}$, the slot list it has recorded is$\;\{s_{1},s_{2},\ldots \ldots ,s_{e}$}. Depending on the number of slots it added, it begins receiving data at any slot within$\;\left\{s_{1},\ldots \ldots ,s_{d+1}\right\}$. If the reception fails, the node will wake up again at the next slot in the slot list to try to receive data packets. The son nodes will stop waking up at other slots when either of the following two conditions is met:

1. The packet is received successfully
2. It senses nothing at one of its own slots$\;\left\{s_{1},\ldots \ldots ,s_{d+1}\right\}$

Initializing process of father node under AAPS scheme can be described by Algorithm 9.

**Algorithm 9** Initialize father node under AAPS scheme1: Son_Count = 0 2: **For** each node of Son_Nodes
**Do**
3:  **For** each awake slot of this node **Do** 4:   Add this slot to father node’s sending slot list 5:  **End for** 6:  Son_Count = Son_Count + 1 7: **End for**

Initializing process of son nodes under AAPS scheme can be described by Algorithm 10.

**Algorithm 10** Initialize son nodes under AAPS scheme1:Max_Interval = 0, Min_Interval = 0, *d* = NumberOfAddedSlots, *i* = 0 2:Min_Interval = (*m* − *d* − 1)/(*d* + 1) 3:**If** (*m* − *d* − 1)mod(*d* + 1)==0 **then** 4: Max_Interval = Min_Interval 5:**End if** 6:**Else** 7: Max_Interval = Min_Interval + 1 8:**End else** 9:**While**
*i* < *d*
**Do** 10: **If** i mod 2==0 **then** 11: Add (OwnSlot + Min_Interval + 1) to awake slot list 12: **End if** 13: **Else** 14: Add (OwnSlot + Max_Interval + 1) to awake slot list 15: **End else** 16: *i* = *i* + 1 17:**End while** 18:**For** each node of Brother_Nodes **Do** 19:   **For** each awake slot of this brother node **Do** 20:   add this awake slot to receiving slot list 21:   **End for** 22:**End for**

Working procedure of father node under AAPS scheme can be described by Algorithm 11.

**Algorithm 11** Broadcast data packet under AAPS scheme1:Success_Count = 0 2:**While** Success_Count < Son_Count **Do** 3:  wait until the next slot in sending slot list 4:  broadcast data packet 5:  **For** each node of Son_Nodes
**Do** 6:   **If** this node receives successfully **then** 7:   Success_Count = Success_Count + 1 8:   **End if** 9:  **End for** 10: **End while**

Working procedure of son nodes under AAPS scheme can be described by Algorithm 12.

**Algorithm 12** Receive data packet under AAPS scheme1: Success_Flag = 0,Receive_Flag = 0 2:**While** Receive_Flag==0 **Do** 3:  wait until next slot in awake slot list 4:  **If** this node receives successfully **then**
5:    Success_Flag = 1, Receive_Flag = 1 6:  **End if** 7:  **Else if** this node receives unsuccessfully **then** 8:   Success_Flag = 0, Receive_Flag = 1 9:  **End else** 10:**End while** 11: **While** Success_Flag==0 **Do** 12:   wait until the next slot in receiving slot list 13:   **If** this node receives successfully **then** 14:    Success_Flag = 1 15:   **End if** 16:**End while**


## 5. Parameter Optimization and Performance Analysis

### 5.1. Calculations of Energy and the Number of Slots that Can Be Added

**Definition 4.**  *Empty delay is the total number of slots between the time the parent is ready and its first attempt to send packets to the son.*

**Definition 5.**  *Transmission delay is the total number of slots remaining in the total delay when empty delay is removed.*

**Figure 6:**
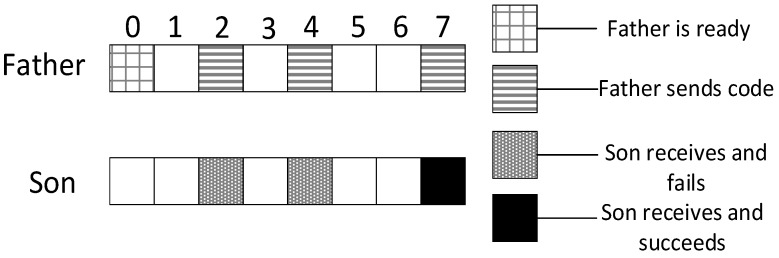
The example of Definitions 4 and 5.

As shown in Figure 6, the parent node is ready at slot 0 and the son node wakes up for the first time at slot 2. According to definition 4, in this example, the empty delay is 2. The son node wakes up for three times each at slot 2, slot 4 and slot 7. It fails at the first two slots and succeeds at the last slot. Therefore, according to definition 5, in this example, the transmission delay is 5.

**Theorem 1.**  *Given the single transmission success rate*$\;P_{trans}$*and the threshold value of total transmission success rate*$\;P_{th}$*, the maximum transmission times is*$\;T_{max}=⎡\frac{log\left(1-P_{th}\right)}{log\left(1-P_{trans}\right)}⎤$.

**Proof.**  $P_{trans}$ represents the success rate of a single transmission, so $\left(1-P_{trans}\right)$ represents the failure rate of a single transmission. Since the packet is sent for$\;T_{max}$ times,$\;{\left(1-P_{trans}\right)}^{T_{max}}$ represents the failure rate after $T_{max}$ transmissions and $1-{\left(1-P_{trans}\right)}^{T_{max}}$ represents the success rate after $T_{max}$ transmissions.

In order to make the success rate greater than the threshold$\;P_{th}$, the following inequality should be satisfied:

$$\;1-{\left(1-P_{trans}\right)}^{T_{max}}\geq P_{th}\;$$

Solve this inequality and we get:

$$T_{max}\geq \frac{\log\left(1-P_{th}\right)}{\log\left(1-P_{trans}\right)}.$$

Obviously, the transmission times is the smallest integer that satisfies this inequality, so:

$$T_{max}=⎡\frac{\log\left(1-P_{th}\right)}{\log\left(1-P_{trans}\right)}⎤.$$

□

**Figure 7:**
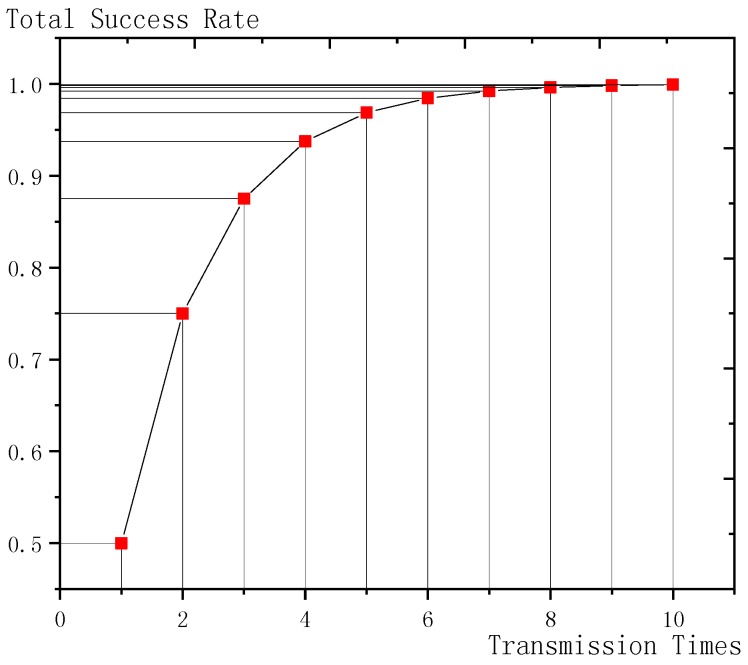
The example of Theorem 1 ($P_{trans\;}$ = 0.5).

As shown in Figure 7, the x-coordinate is the transmission times and the y-coordinate is the total transmission success rate. As the transmission times increases, the total transmission success rate increases. When the single transmission success rate is set to 0.5, as shown in the figure, when the transmission times are over 6, the total transmission success rate raised by the increase of transmission times is very small but it consumes more energy. Therefore, setting the maximum transmission times can reduce energy consumption under the condition that the total success rate is almost not affected.

**Theorem 2.**  *Given the transmission layer, then the number of the total forwarded data packets is*$\;\frac{n\left(R^{2}-r^{2}\right)-4nr^{2}\left(i^{2}-i\right)}{R^{2}}$.

**Figure 8:**
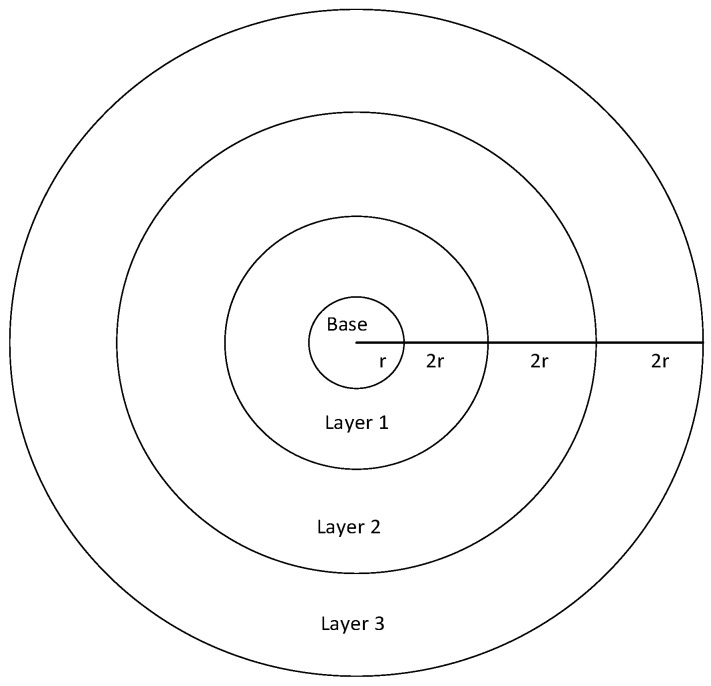
Networks layer model.

**Proof.**  As shown in Figure 8, the inner radius of the annular transmission layer i is (2i−1)r and the external radius is (2i+1)r, therefore, according to the formula of circular area, the area of transmission layer i is$\;\pi \times \left({\left(\left(2i+1\right)r\right)}^{2}-{\left(\left(2i-1\right)r\right)}^{2}\right)$, since the node density is$\;\rho =\frac{n}{\pi R^{2}}$, after simplification, the number of nodes in this layer is$\;\frac{8nr^{2}}{R^{2}}\times i$.

Since the radius of the whole area with nodes is R, there are$\;\frac{R-r}{2r}-\left(R-r\right)\%2r\;$complete transmission layers and an incomplete one with the width of (R−r)%2r. Since there is a total of i−1 layers before the $i_{th}$ layer, the total number of nodes in these i−1 layers is$\;\sum_{j=1}^{j=i-1}\frac{8nr^{2}}{R^{2}}\times j$, simplified to$\;\frac{4nr^{2}\left(i^{2}-i\right)}{R^{2}}$. As all the layers form a ring with an inner radius of r and an external radius of R, the total number of nodes is$\;\frac{n\left(R^{2}-r^{2}\right)}{R^{2}}$. Therefore, the total number of data packets forwarded by the $i_{th}$ layer is$\;\frac{n\left(R^{2}-r^{2}\right)-4nr^{2}\left(i^{2}-i\right)}{R^{2}}$. □

**Figure 9:**
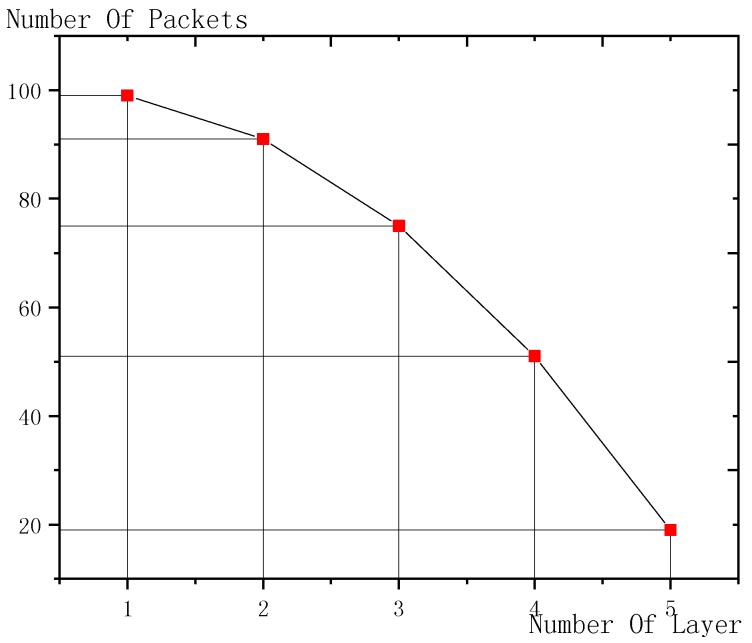
Comparison of the packets forwarded by each layer (*n* = 100, R = 100, r = 10).

As shown in Figure 9, the *x*-coordinate is the transmission layer and the *y*-coordinate is the number of forwarded data packets. From the figure, it can be concluded that, the further layer is away from the sink node, the less data packets it is responsible to forward, which is because: the nodes near the sink node need to forward not only the data of its own but also all the data from external layers. Therefore, the nearer node is from the sink node, the bigger is the number of data forwarded; the further node is away from the sink node, the smaller is the number of data forwarded.

**Theorem 3.**  *Given the transmission layer*i, *supposing the data packets generated by each node are of the same size and the time of collecting and forwarding data is as long as a cycle, then, there is energy surplus, which is*$\frac{\left(i-1\right)\left(E_{trans}+E_{receive}\right)}{2}$*per node in layer*i*. The additional energy can be used to add slots, ultimately,*$\frac{9}{2}\left(i-1\right)$*slots can be added.*

**Proof.**  According to Theorem 2, given the layer i, the number of forwarded packets is$\;\frac{n\left(R^{2}-r^{2}\right)-4nr^{2}\left(i^{2}-i\right)}{R^{2}}$, taking the derivative of which, we get$\;\frac{-4nr^{2}\left(2i-1\right)}{R^{2}}$. Since i≥1, the derivative is constantly smaller than 0. Therefore, the further layer is away from the root, the less packets it forwards. When i=1, maximum packets are forwarded, which is$\;\frac{n\left(R^{2}-r^{2}\right)}{R^{2}}$. Thus, compared with the maximum, each layer forwarded $\frac{4nr^{2}\left(i^{2}-i\right)}{R^{2}}$ less packets. Since the energy consumpted to process the packet is$\;E_{trans}+E_{receive}$, the energy surplus of this layer is$\;\left(E_{trans}+E_{receive}\right)$. Because the number of nodes in this layer is$\;\frac{8nr^{2}}{R^{2}}\times i$, the energy left over for each node in this layer is: $\frac{\left(i-1\right)\left(E_{trans}+E_{receive}\right)}{2}$. Thus, the number of slots added at most is:

$$\frac{\left(i-1\right)\left(E_{trans}+E_{receive}\right)}{2E_{awake}}=\frac{9}{2}\left(i-1\right).\;$$

□

**Theorem 4.**  *Since son node wakes up randomly at one of the slots in a cycle, the expected empty delay is* $\frac{m-1}{2}$ *. Using AAPS, if*  d  *slots are added, the expected empty delay is accordingly* $\;\frac{\frac{m}{d+1}-1}{2}$ *. The more slots are added, the smaller is the expected empty delay. At most*  m−1 *slots can be added, which can achieve the smallest empty delay, which is 0.*

**Proof.**  At first, son node only wakes up at one of m slots in a cycle, while the parent node can be ready to send the packet at any slot. Supposing parent node is ready at$\;s_{father}$ and son node wake up at$\;s_{son}$, the empty delay is thus:

$$\{\begin{array}{c} s_{son}-s_{father}\;,\;s_{son}\geq s_{father}\\ s_{son}+m-s_{father}\;,\;s_{son}<s_{father} \end{array}.$$

The expectation of which is $\frac{1}{\;m}\sum_{s_{son}=0}^{s_{son}=m-1}\left(\frac{1}{m}\sum_{s_{father=0}}^{s_{father=}s_{son}}\left(s_{son}-s_{father}\right)+\;\frac{1}{m}\sum_{s_{father=s_{son}+1}}^{s_{father}=m-1}\left(s_{son}+m-s_{father}\right)\right)$.

After simplification, we get$\;\frac{m-1}{2}$. After d slots are added, similar to the deduction above, the expectation of empty delay is$\;\frac{\frac{m}{d+1}-1}{2}$, taking the derivative of which, we get$\;-\frac{m}{2{\left(d+1\right)}^{2}}$, which is constantly smaller than 0. Thus, the more are the slots added, the smaller is the expectation of empty delay. Since d≤m−1, the minimum value 0 is reached when d=m−1. In this circumstance, son node never sleeps, therefore parent node can broadcast the packet anytime it wishes. □

**Figure 10:**
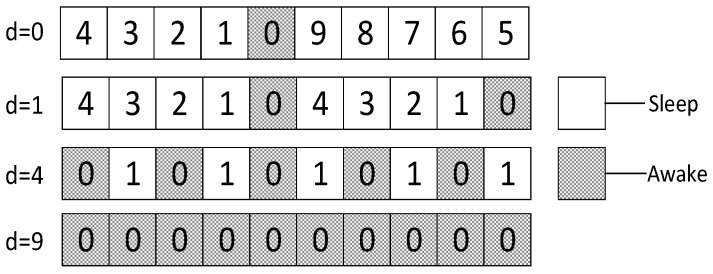
Example of the effect of added slots on empty delay (*m* = 10).

Shown in Figure 10 is the example of the effect of added slots on empty delay. The number in the figure denotes the empty delay when parent node is ready at this slot. When *d* = 0, namely when no slots are added, parent node has to wait at most 9 slots before son node wakes up and the average delay is 9/2; with one slot added, the maximum empty delay has been reduced to 4 and the average of delay is 2; with 4 slots added, the maximum empty delay has been reduced to 1 and the average delay is 1/2; with 9 slots added, the cycle is completely covered by son nodes and the empty delay is 0.

**Figure 11:**
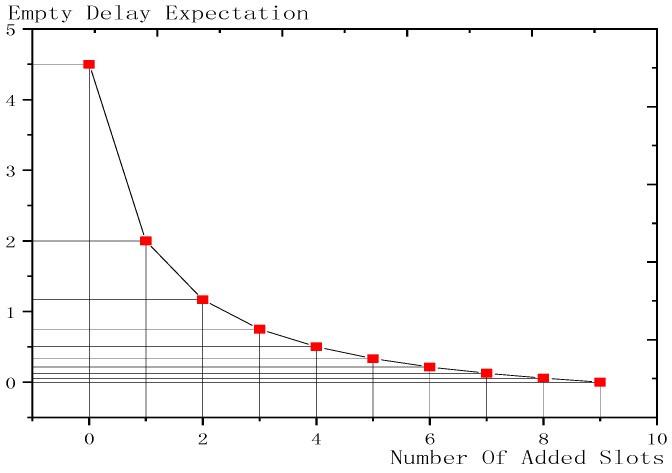
The trend chart of the effect of added slots on empty delay (*m* = 10).

Shown in Figure 11 is the change trend of the effect of added slots on empty delay. The abscissa is the number of added slots and the ordinate is the corresponding empty delay. The more slots are added, the smaller is the expectation of empty delay, which is finally reduced to 0. At first, increasing the number of slots has a very good effect. Adding one slot reduces the delay expectation by half but as the number of slots increases, the reduction becomes slower and slower.

**Theorem 5.**  *The minimum transmission delay exists.*

**Proof.**  According to Definition 5, in networks without transmission failure, the transmission delay is 0, while in networks with transmission failure, the transmission delay is mostly decided by the slot at which parent node is ready. Apparently, when parent node broadcasts at every slot and son node wakes up at every slot, the transmission delay is the minimum. □

**Figure 12:**
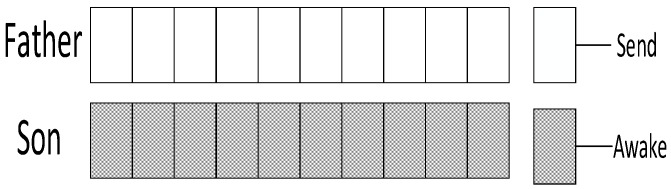
Example of slots when transmission is the minimum (*m* = 10).

Shown in Figure 12 is the distribution of slots when nodes have the minimum transmission delay. Since the node wakes up at each slot, when the node fails to receive the code, it can immediately wake up at the next slot and receive the code again, thus obtaining the shortest transmission delay.

**Theorem 6.**  *With AAPS, minimum transmission delay can be achieved. Given the number of son nodes (denoted as* j*), at the worst condition, a total of* m−1 *slots should be added, on average*$\;\frac{m-1}{j}\;$*slots should be added by each node. At the best condition, a total of* m−j *slots should be added, on average*$\;\frac{m}{j}\;$*slots should be added by each node. Let* i *denote the transmission layer, when*$\;i\geq \frac{2\left(m-1\right)}{9j}+1$, *the energy surplus is enough to guarantee minimum transmission delay.*

**Figure 13:**
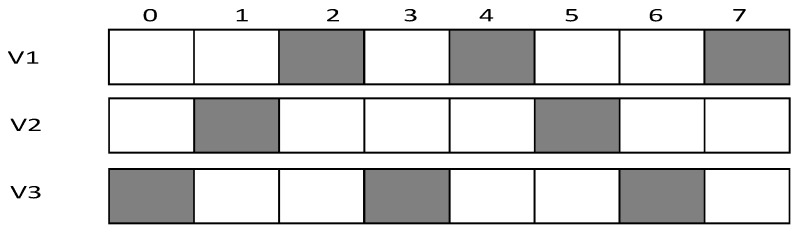
Minimum transmission delay model.

**Proof.**  According to Theorem 5, the minimum transmission delay exists. The distribution of slots when minimum transmission delay is achieved is shown in Figure 12. With AAPS, the slots are added and when node starts receiving code, since it can make use of brother nodes’ slots, the expectation of transmission delay is decided by the single transmission success rate and the coverage of slots in cycle. When the slots of all the son nodes cover the cycle, as shown in Figure 13, the minimum transmission delay is achieved. In this circumstance, adding more slots can no longer reduce transmission delay. According to Theorem 4, when the total number of added slots is smaller than m−1, the more slots are added, the smaller is the empty delay.

**Figure 14:**
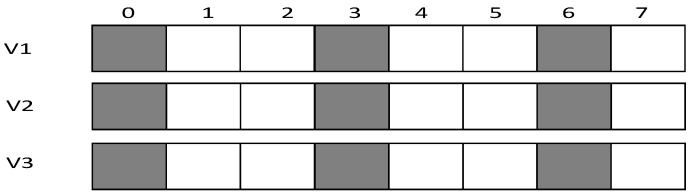
Model of son nodes’ having the same slots.

In AAPS, since the node makes use of brother nodes’ slots, it can wake up as frequently as possible in a cycle to receive data packet and reduce transmission delay. However, when all the son nodes share the same slots, as shown in Figure 14, they can not use each other’s slots to try receiving code, therefore this is the worst case for AAPS.

Assuming there are j son ndoes, in order to completely cover all the slots in a cycle, in the worst scenario described above, since there are m slots, all the nodes have to add a total of m−1 slots, $\frac{m-1}{j}\;\mathrm{slots}\;$per node. In the best scenario, where all the son nodes’ slots are different, all the nodes only need to add m−j slots, averagely $\frac{m-j}{j}$ per node. Since the added slots can be at the same position, all the node should have one redundant slot added, therefore, they should add $\frac{m}{j}$ slots individually, so that minimum transmission delay can be reached at most cases.

According to Theorem 3, given the layer i, at most$\;\frac{9}{2}\left(i-1\right)\;$slots can be added by each node in this layer. Assuming there are j son nodes, as proved before, at most$\;\frac{m-1}{j}$ slots need to be added to guarantee minimum transmission delay. Therefore, when$\;\frac{9}{2}\left(i-1\right)\geq \frac{m-1}{j}$, minimum transmission delay can be achieved, the inequality above can be simplified to$\;i\geq \frac{2\left(m-1\right)}{9j}+1$. □

### 5.2. Delay Calculation

**Theorem 7.**  *During the initialization, supposing there are* k *son nodes, then the number of slots with nodes waking up is expected to be*$\;m\times \left(1-{\left(1-\frac{1}{m}\right)}^{k}\right)$*and on average*$\frac{k}{m\times \left(1-{\left(1-\frac{1}{m}\right)}^{k}\right)}\;$*nodes wake up in each of these slots, the expected slots node can use to add additional awake slot in a cycle is*$\;\frac{m\times \left(1-{\left(1-\frac{1}{m}\right)}^{k}\right)-1}{2}$*, the slot with node waking up for the first time is expected to be*$\frac{m-1}{2}$*and the average interval is*$\;\frac{{\left(1-\frac{1}{m}\right)}^{k}}{m\times \left(1-{\left(1-\frac{1}{m}\right)}^{k}\right)}$.

**Proof.**  Let f(k) denote the number of slots with node waking up after the $k_{th}\;$node is added. Obviously, f(1)=1. If the added node wakes up at the slot used by previous nodes, then the number of used slots is not changed, otherwise, the number of added slots should be added 1. Therefore,$\;f\left(k+1\right)=\frac{f\left(k\right)}{m}\times f\left(k\right)+\frac{m-f\left(k\right)}{m}\times \left(f\left(k\right)+1\right)$, after simplification, we can get$\;f\left(k+1\right)=\frac{m-1}{m}\times f\left(k\right)+1$. Since f(1)=1, using mathematical induction, we can get$\;f\left(k\right)=m\times \left(1-{\left(1-\frac{1}{m}\right)}^{k}\right)$. Because there are k son nodes,$\;\frac{k}{f\left(k\right)}=\frac{k}{m\times \left(1-{\left(1-\frac{1}{m}\right)}^{k}\right)}$ which is the average number of nodes waking up in a slot. According to the symmetry, the expected number of slots when parent node broadcasts after every node is the same with that before every node and the sum of them is the expected number of slots with node waking up minus one. Therefore, the number of slots when parent node broadcasts after every node is $\frac{m\times \left(1-{\left(1-\frac{1}{m}\right)}^{k}\right)-1}{2}$. Since the probability of waking up in a slot is equal, in a cycle, the slot with node waking up for the first time is expected to be $\frac{1}{m}\sum_{i=0}^{i=m-1}i=\frac{m-1}{2}$. Since there are$\;m\times \left(1-{\left(1-\frac{1}{m}\right)}^{k}\right)$ awake slots in a cycle, there are$\;{\left(1-\frac{1}{m}\right)}^{k}$ empty slots and the average interval is$\;\frac{{\left(1-\frac{1}{m}\right)}^{k}}{m\times \left(1-{\left(1-\frac{1}{m}\right)}^{k}\right)}$. □

**Figure 15:**
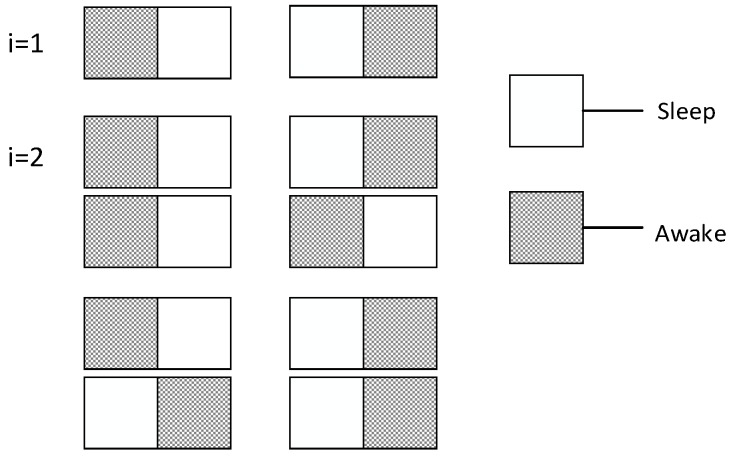
Example of Theorem 7 (*m* = 2, *i* = 2).

Figure 15 shows an example of Theorem 7. Because there are only two slots, there are only two possible cases of node’s waking up as shown in the figure where *i* = 1. When *i* = 2, there are four possible cases as shown in the figure. When the number of slots is 2 and the number of node is 1, the expectation of total number of slots is 3/2.

**Figure 16:**
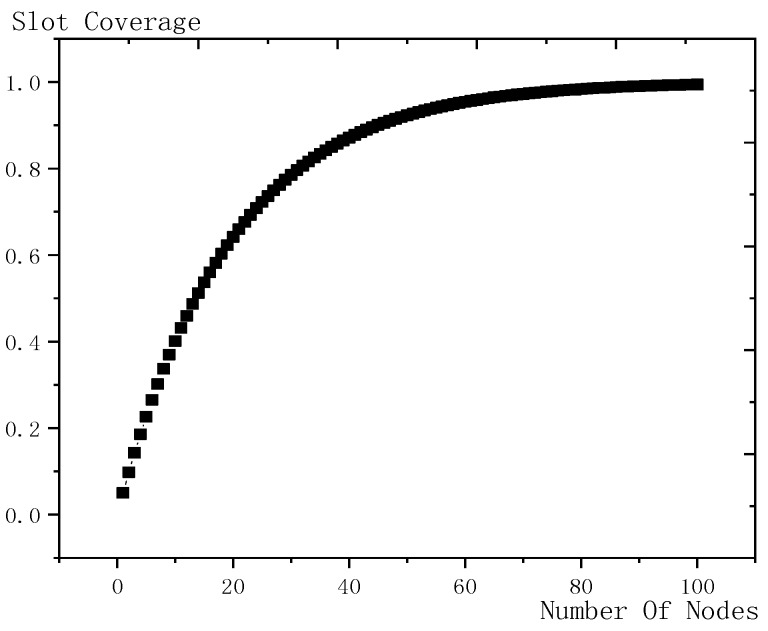
The influence trend of the number of nodes on slot coverage (*m* = 20).

Shown in Figure 16 is the influence trend of the number of son nodes on slot coverage, the abscissa is the number of son nodes and the ordinate is the corresponding slot coverage. When the number of son nodes increases, the coverage increases. Initially, the coverage increases rapidly and with the increase of the number of son nodes, the coverage growth slows down. This is because, as the number of son nodes increases, the slot coverage increases, the new nodes are more likely to share slot with the existing nodes, so the improvement effect on slot coverage is very small.

**Theorem 8.**  *In traditional method, the expected delay is*$\;\frac{m-1}{2}+\sum_{j=1}^{j=T_{max}}\left(P_{trans}\;\times {\left(1-P_{trans}\right)}^{j-1}\times \left(j-1\right)\times m\right)$.

**Proof.**  Assuming that parent node is ready at$\;s_{father}$ and son node wakes up at$\;s_{son}$, the empty delay is $\{\begin{array}{c} s_{son}-s_{father}\;,\;s_{son}\geq s_{father}\\ s_{son}+m-s_{father}\;,\;\;s_{son}<s_{father} \end{array}$, similar to the proof in Theorem 4, the expected empty delay is $\frac{m-1}{2}$. The expected transmission delay is$\;\sum_{j=1}^{j=T_{max}}\left(P_{trans}\;\times {\left(1-P_{trans}\right)}^{j-1}\times \left(j-1\right)\times m\right)$. Therefore, the expected total delay is $\frac{m-1}{2}+\sum_{j=1}^{j=T_{max}}\left(P_{trans}\;\times {\left(1-P_{trans}\right)}^{j-1}\times \left(j-1\right)\times m\right)$. □

**Theorem 9.**  *In the worst case, the IFAS algorithm is equivalent to the traditional algorithm; in other cases, after this son node, it is assumed that there is a total of*  i  *slots when nodes wake up to receive data and the expectation of delay of each node is*

$$\;\{\begin{array}{c} \frac{m-1}{2}+\\ \overset{j=T_{max}}{\underset{j=1}{\sum }}\left(P_{trans}\;\times {\left(1-P_{trans}\right)}^{j-1}\times \left(s_{j}-s_{son}\right)\right)\\ ,i\geq T_{max}\\ \frac{m-1}{2}+\\ \overset{j=T_{max}}{\underset{j=1}{\sum }}\left(\begin{array}{c} P_{trans}\;\times {\left(1-P_{trans}\right)}^{j-1}\times \\ \left(\left(s_{j\%\left(i+1\right)-1}-s_{son}\right)+m\times \frac{\left(j-1\right)-\left(j-1\right)\%\left(i+1\right)}{i+1}\right) \end{array}\right)\\ ,i<T_{max} \end{array}\;$$

**Proof.**  Since the principle of IFAS shortening delay is to try to receive data packets by using the slots of brother nodes after the failure of receiving, when the transmission success rate of data packets is 100%, it will not fail to receive and the transmission delay cannot be reduced. Therefore, it is equivalent to receiving data packets only at the initial slot, namely the traditional algorithm.

In other cases, assuming that parent node is ready at$\;s_{father}$ and son node wakes up at$\;s_{son}$ and there is a total of i slots when nodes wake up to receive data after this son node, $\left\{s_{1},s_{2},\ldots \ldots ,s_{i}\right\}$. Write$\;s_{son}\;$as$\;s_{0}$.

The empty delay is

$\{\begin{array}{c} s_{son}-s_{father}\;,s_{son}\geq s_{father}\\ s_{son}+m-s_{father}\;,s_{son}<s_{father} \end{array}$, similar to the proof in Theorem 4, the expected empty delay is$\;\frac{m-1}{2}$.

And the expectation of transmission delay is$\{\begin{array}{c} \sum_{j=1}^{j=T_{max}}\left(P_{trans}\;\times {\left(1-P_{trans}\right)}^{j-1}\times \left(s_{j}-s_{son}\right)\right)\\ ,i\geq T_{max}\\ \sum_{j=1}^{j=T_{max}}\left(\begin{array}{c} P_{trans}\;\times {\left(1-P_{trans}\right)}^{j-1}\times \\ \left(\left(s_{j\%\left(i+1\right)-1}-s_{son}\right)+m\times \frac{\left(j-1\right)-\left(j-1\right)\%\left(i+1\right)}{i+1}\right) \end{array}\right)\\ ,i<T_{max} \end{array}$.

Thus, the expected total delay is $\{\begin{array}{c} \frac{m-1}{2}+\sum_{j=1}^{j=T_{max}}\left(P_{trans}\;\times {\left(1-P_{trans}\right)}^{j-1}\times \left(s_{j}-s_{son}\right)\right)\\ ,i\geq T_{max}\\ \frac{m-1}{2}+\sum_{j=1}^{j=T_{max}}\left(\begin{array}{c} P_{trans}\;\times {\left(1-P_{trans}\right)}^{j-1}\times \\ \left(\left(s_{j\%\left(i+1\right)-1}-s_{son}\right)+m\times \frac{\left(j-1\right)-\left(j-1\right)\%\left(i+1\right)}{i+1}\right) \end{array}\right)\\ ,i<T_{max} \end{array}$. □

**Theorem 10.**  *BTAS can always get better results than IFAS and always get better results than traditional algorithm. Supposing there are i + 1 slots with node waking up, the expectation of delay of the last node is*

$$\;\frac{s_{i}-s_{0}}{m}\times \sum_{j=2}^{j=T_{max}}\left(\begin{array}{c} P_{trans}\;\times {\left(1-P_{trans}\right)}^{j-1}\times \\ \left(\begin{array}{c} \left(m-s_{i}+s_{\left(j-2\right)\%\left(i+1\right)}\right)+\\ m\times \left(\frac{\left(j-2\right)-\left(j-2\right)\%\left(i+1\right)}{i+1}\right) \end{array}\right) \end{array}\right)+\frac{m-s_{i}+s_{0}}{m}\times \;\;\sum_{j=1}^{j=T_{max}}\left(\begin{array}{c} P_{trans}\;\times {\left(1-P_{trans}\right)}^{j-1}\times \\ \left(\left(s_{j\%\left(i+1\right)-1}-s_{0}\right)+m\times \frac{\left(j-1\right)-\left(j-1\right)\%\left(i+1\right)}{i+1}\right) \end{array}\right)+\frac{{\left(s_{i}-s_{0}\right)}^{2}+{\left(m-s_{i}+s_{0}\right)}^{2}}{2m}.$$

**Proof.**  According to Theorem 9, the IFAS algorithm is superior to the traditional algorithm in most cases. In only a few cases, it can achieve the same effect as the traditional algorithm. Compared with the IFAS algorithm, BTAS optimizes the nodes that wake up last and cannot use the slots of brother nodes, greatly reducing the expectation of its delay, so it can always get better results than IFAS and always get better results than traditional algorithm.

Since the last node in the IFAS algorithm cannot use the brother nodes’ slots, assuming that parent node is ready at *s_father_* and son node wakes up at *s_son_*, the expectation of transmission is

$$\{\begin{array}{c} \left(s_{son}-s_{father}\right)\times P_{trans}+\\ \sum_{i=1}^{i=T_{max}-1}\left(P_{trans}\;\times {\left(1-P_{trans}\right)}^{i-1}\times m\times i\right)\\ ,s_{son}\geq s_{father}\\ \left(s_{son}+m-s_{father}\right)\times P_{trans}+\\ \sum_{i=1}^{i=T_{max}-1}\left(P_{trans}\;\times {\left(1-P_{trans}\right)}^{i-1}\times m\times i\right)\;\\ \;,s_{son}<s_{father} \end{array}.$$

the expectation of total delay is $\left\{ \begin{array}{c} \frac{m-1}{2}+\left(s_{son}-s_{father}\right)\times P_{trans}+\\ \sum_{i=1}^{i=T_{max}-1}\left(P_{trans}\;\times {\left(1-P_{trans}\right)}^{i-1}\times m\times i\right)\\ ,s_{son}\geq s_{father}\\ \frac{m-1}{2}+\left(s_{son}+m-s_{father}\right)\times P_{trans}+\\ \sum_{i=1}^{i=T_{max}-1}\left(P_{trans}\;\times {\left(1-P_{trans}\right)}^{i-1}\times m\times i\right)\\ ,s_{son}<s_{father} \end{array}\right.$.

With BTAS, this node share$\;s_{first}\;$with the first node. Supposing there are i+1 slots with node waking up, $\left\{s_{0},s_{1},\ldots \ldots ,s_{i}\right\}$, then$\;s_{first}=s_{0}$, $s_{son}=s_{i}$, the expectation of empty delay is $\;\frac{s_{i}-s_{0}}{2}\times \frac{s_{i}-s_{0}}{m}+\frac{m-s_{i}+s_{0}}{2}\times \frac{m-s_{i}+s_{0}}{m}=\frac{{\left(s_{i}-s_{0}\right)}^{2}+{\left(m-s_{i}+s_{0}\right)}^{2}}{2m}$ and the expected transmission delay is$\frac{s_{i}-s_{0}}{m}\times \sum_{j=2}^{j=T_{max}}\left(\begin{array}{c} P_{trans}\;\times {\left(1-P_{trans}\right)}^{j-1}\times \\ \left(\begin{array}{c} \left(m-s_{i}+s_{\left(j-2\right)\%\left(i+1\right)}\right)+\\ m\times \left(\frac{\left(j-2\right)-\left(j-2\right)\%\left(i+1\right)}{i+1}\right) \end{array}\right) \end{array}\right)+\frac{m-s_{i}+s_{0}}{m}\times \sum_{j=1}^{j=T_{max}}\left(\begin{array}{c} P_{trans}\;\times {\left(1-P_{trans}\right)}^{j-1}\times \\ \left(\left(s_{j\%\left(i+1\right)-1}-s_{0}\right)+m\times \frac{\left(j-1\right)-\left(j-1\right)\%\left(i+1\right)}{i+1}\right) \end{array}\right)$ the expected total delay is $\frac{s_{i}-s_{0}}{m}\times \sum_{j=2}^{j=T_{max}}\left(\begin{array}{c} P_{trans}\;\times {\left(1-P_{trans}\right)}^{j-1}\times \\ \left(\begin{array}{c} \left(m-s_{i}+s_{\left(j-2\right)\%\left(i+1\right)}\right)+\\ m\times \left(\frac{\left(j-2\right)-\left(j-2\right)\%\left(i+1\right)}{i+1}\right) \end{array}\right) \end{array}\right)+\frac{m-s_{i}+s_{0}}{m}\times \sum_{j=1}^{j=T_{max}}\left(\begin{array}{c} P_{trans}\;\times {\left(1-P_{trans}\right)}^{j-1}\times \\ \left(\left(s_{j\%\left(i+1\right)-1}-s_{0}\right)+m\times \frac{\left(j-1\right)-\left(j-1\right)\%\left(i+1\right)}{i+1}\right) \end{array}\right)+\frac{{\left(s_{i}-s_{0}\right)}^{2}+{\left(m-s_{i}+s_{0}\right)}^{2}}{2m}$. □

**Theorem 11.**  *With AAPS, after the working cycle is fully covered, the expected total delay is*$\;\frac{\frac{m}{d+1}-1}{2}+\sum_{i=0}^{i=T_{max}-1}\left(P_{trans}\times {\left(1-P_{trans}\right)}^{i}\times i\right)$.

**Proof.**  According to Theorem 4, after d slots are added, the expected empty delay is $\frac{\frac{m}{d+1}-1}{2}$. Assuming that the cycle is fully covered, the expectation of transmission delay is$\;\sum_{i=0}^{i=T_{max}-1}\left(P_{trans}\times {\left(1-P_{trans}\right)}^{i}\times i\right)$. Thus, the expected total delay is$\;\frac{\frac{m}{d+1}-1}{2}+\sum_{i=0}^{i=T_{max}-1}\left(P_{trans}\times {\left(1-P_{trans}\right)}^{i}\times i\right)$. □

**Premise.**  According to Theorem 7, the distribution of nodes’ slots is decided by the number of slots in a cycle and son nodes, therefore, in the following analysis, it is assumed that m and the number of son nodes (denoted as k) is constant. Therefore, there is a total of $\frac{m\times \left(1-{\left(1-\frac{1}{m}\right)}^{k}\right)-1}{2}$ slots that can be used to receive packet after failure. According to Theorem 8 and Theorem 9, the expected delay is affected by the number of slots that can be used after failure and$\;T_{max}$. In practice, the number of son nodes is usually big, thus making the number of useful slots bigger than$\;T_{max}$, namely$\;\frac{m\times \left(1-{\left(1-\frac{1}{m}\right)}^{k}\right)-1}{2}\;\geq {\;T}_{max}$. Therefore, in the following analysis, it is assumed that $\;T_{max}$ and $P_{trans}$ are constant and m, k,$\;T_{max}$ satisfy the inequality stated above.

**Theorem 12.**  *Suppose the number of son nodes is* k*. The expected delay for all the nodes under traditional algorithm is*$\;\frac{m-1}{2}+m\times \left(\frac{1}{P_{trans}}-1\right)-m\times \left(T_{max}+\frac{1}{P_{trans}}-1\right)\times {\left(1-P_{trans}\right)}^{T_{max}}$*. The expected delay for all the nodes under IFAS is*$\;\frac{m-1}{2}+\left(\frac{{\left(1-\frac{1}{m}\right)}^{k}}{m\times \left(1-{\left(1-\frac{1}{m}\right)}^{k}\right)}+1\right)\times \left(\frac{1}{P_{trans}}-1\right)-\left(\frac{{\left(1-\frac{1}{m}\right)}^{k}}{m\times \left(1-{\left(1-\frac{1}{m}\right)}^{k}\right)}+1\right)\times \left(T_{max}+\frac{1}{P_{trans}}-1\right)\times {\left(1-P_{trans}\right)}^{T_{max}}$*. The expected delay for the last node under BTAS is*$\left(\frac{{\left(1-\frac{1}{m}\right)}^{k}}{m\times \left(1-{\left(1-\frac{1}{m}\right)}^{k}\right)}+1\right)\times \left(\frac{1}{P_{trans}}-1\right)-\left(\frac{{\left(1-\frac{1}{m}\right)}^{k}}{m\times \left(1-{\left(1-\frac{1}{m}\right)}^{k}\right)}+1\right)\times \left(T_{max}+\frac{1}{P_{trans}}-1\right)\times {\left(1-P_{trans}\right)}^{T_{max}}+\frac{{\left(\frac{{\left(1-\frac{1}{m}\right)}^{k}}{m\times \left(1-{\left(1-\frac{1}{m}\right)}^{k}\right)}+1\right)}^{2}+{\left(m-\frac{{\left(1-\frac{1}{m}\right)}^{k}}{m\times \left(1-{\left(1-\frac{1}{m}\right)}^{k}\right)}-1\right)}^{2}}{2m}$*. The expected delay for all the nodes under AAPS is*$\;\frac{k-1}{2}+\left(\frac{1}{P_{trans}}-1\right)-\left(T_{max}+\frac{1}{P_{trans}}-1\right)\times {\left(1-P_{trans}\right)}^{T_{max}}$.

**Proof.**  According to Theorem 8, the delay under traditional algorithm is$\;\frac{m-1}{2}+\sum_{j=1}^{j=T_{max}}\left(P_{trans}\;\times {\left(1-P_{trans}\right)}^{j-1}\times \left(j-1\right)\times m\right)$. Therefore, after simplification, we get$\;\frac{m-1}{2}+m\times \left(\frac{1}{P_{trans}}-1\right)-m\times \left(T_{max}+\frac{1}{P_{trans}}-1\right)\times {\left(1-P_{trans}\right)}^{T_{max}}$.

According to Theorem 9, the delay under IFAS algorithm is$\;\frac{m-1}{2}+\sum_{j=1}^{j=T_{max}}\left(P_{trans}\;\times {\left(1-P_{trans}\right)}^{j-1}\times \left(s_{j}-s_{son}\right)\right)$ and according to Theorem 7, a node is expected to wake up at$\;\frac{m-1}{2}$ and the average interval is$\;\frac{{\left(1-\frac{1}{m}\right)}^{k}}{m\times \left(1-{\left(1-\frac{1}{m}\right)}^{k}\right)}$, thus, $s_{son}=\frac{m-1}{2},\;s_{j}-s_{son}=\frac{j\times {\left(1-\frac{1}{m}\right)}^{k}}{m\times \left(1-{\left(1-\frac{1}{m}\right)}^{k}\right)}$. Therefore, after simplication, the expected delay under IFAS algorithm is$\;\frac{m-1}{2}+\left(\frac{{\left(1-\frac{1}{m}\right)}^{k}}{m\times \left(1-{\left(1-\frac{1}{m}\right)}^{k}\right)}+1\right)\times \left(\frac{1}{P_{trans}}-1\right)-\left(\frac{{\left(1-\frac{1}{m}\right)}^{k}}{m\times \left(1-{\left(1-\frac{1}{m}\right)}^{k}\right)}+1\right)\times \left(T_{max}+\frac{1}{P_{trans}}-1\right)\times {\left(1-P_{trans}\right)}^{T_{max}}$.

According to Theorem 10, assuming there is a total of *i* + 1 slots with node waking up, the delay of the last node is expected to be $\frac{s_{i}-s_{0}}{m}\times \sum_{j=2}^{j=T_{max}}\left(\begin{array}{c} P_{trans}\;\times {\left(1-P_{trans}\right)}^{j-1}\times \\ \left(\begin{array}{c} \left(m-s_{i}+s_{\left(j-2\right)\%\left(i+1\right)}\right)+\\ m\times \left(\frac{\left(j-2\right)-\left(j-2\right)\%\left(i+1\right)}{i+1}\right) \end{array}\right) \end{array}\right)+\frac{m-s_{i}+s_{0}}{m}\times \sum_{j=1}^{j=T_{max}}\left(\begin{array}{c} P_{trans}\;\times {\left(1-P_{trans}\right)}^{j-1}\times \\ \left(\left(s_{j\%\left(i+1\right)-1}-s_{0}\right)+m\times \frac{\left(j-1\right)-\left(j-1\right)\%\left(i+1\right)}{i+1}\right) \end{array}\right)+\frac{{\left(s_{i}-s_{0}\right)}^{2}+{\left(m-s_{i}+s_{0}\right)}^{2}}{2m}$. Because$\;\frac{m\times \left(1-{\left(1-\frac{1}{m}\right)}^{k}\right)-1}{2}\geq T_{max}$, thus after simplification, the formular above can be converted to $\frac{s_{i}-s_{0}}{m}\times \sum_{j=2}^{j=T_{max}}\left(\begin{array}{c} P_{trans}\;\times {\left(1-P_{trans}\right)}^{j-1}\times \\ \left(m-s_{i}+s_{j-2}\right) \end{array}\right)+\frac{m-s_{i}+s_{0}}{m}\times \sum_{j=1}^{j=T_{max}}\left(\begin{array}{c} P_{trans}\;\times {\left(1-P_{trans}\right)}^{j-1}\times \\ \left(s_{j-1}-s_{0}\right) \end{array}\right)+\frac{{\left(s_{i}-s_{0}\right)}^{2}+{\left(m-s_{i}+s_{0}\right)}^{2}}{2m}$. Since the number of average empty slots is$\;\frac{{\left(1-\frac{1}{m}\right)}^{k}}{m\times \left(1-{\left(1-\frac{1}{m}\right)}^{k}\right)}$, the delay of the last node under BTAS algorithm is expected to be$\;\left(\frac{{\left(1-\frac{1}{m}\right)}^{k}}{m\times \left(1-{\left(1-\frac{1}{m}\right)}^{k}\right)}+1\right)\times \left(\frac{1}{P_{trans}}-1\right)-\left(\frac{{\left(1-\frac{1}{m}\right)}^{k}}{m\times \left(1-{\left(1-\frac{1}{m}\right)}^{k}\right)}+1\right)\times \left(T_{max}+\frac{1}{P_{trans}}-1\right)\times {\left(1-P_{trans}\right)}^{T_{max}}+\frac{{\left(\frac{{\left(1-\frac{1}{m}\right)}^{k}}{m\times \left(1-{\left(1-\frac{1}{m}\right)}^{k}\right)}+1\right)}^{2}+{\left(m-\frac{{\left(1-\frac{1}{m}\right)}^{k}}{m\times \left(1-{\left(1-\frac{1}{m}\right)}^{k}\right)}-1\right)}^{2}}{2m}$.

According to Theorem 11, the expected delay of node under AAPS algorithm is$\;\frac{\frac{m}{d+1}-1}{2}+\sum_{i=0}^{i=T_{max}-1}\left(P_{trans}\times {\left(1-P_{trans}\right)}^{i}\times i\right)$ and according to Theorem 6, the number of added slots is expected to be$\;\frac{m}{k}-1$, thus after simplification, the delay under AAPS algorithm is expected to be$\;\frac{k-1}{2}+\left(\frac{1}{P_{trans}}-1\right)-\left(T_{max}+\frac{1}{P_{trans}}-1\right)\times {\left(1-P_{trans}\right)}^{T_{max}}$. □

**Theorem 13.**  *Suppose the number of son nodes is* k. *Then compared with the traditional algorithm, IFAS improves the transmission delay by*$\;1-\frac{{\left(1-\frac{1}{m}\right)}^{k}+m\times \left(1-{\left(1-\frac{1}{m}\right)}^{k}\right)}{m^{2}\times \left(1-{\left(1-\frac{1}{m}\right)}^{k}\right)}$*, while AAPS improves the transmission delay by*$\;1-\frac{1}{m}\;$.

**Proof.**  The delay can be divided into empty delay and transmission delay. After previous analysis, we can get that the empty delay of traditional delay is equivalent to that of IFAS, which is $\frac{m-1}{2}$, while the empty delay of AAPS is $\frac{k-1}{2}$, improved by $1-\frac{k-1}{m-1}$, compared with traditional algorithm and IFAS. Compared with the traditional algorithm, the transmission delay is improved by $1-\frac{{\left(1-\frac{1}{m}\right)}^{k}+m\times \left(1-{\left(1-\frac{1}{m}\right)}^{k}\right)}{m^{2}\times \left(1-{\left(1-\frac{1}{m}\right)}^{k}\right)}\;$under IFAS scheme. Compared with the traditional algorithm, the transmission delay is improved by $1-\frac{1}{m}\;$under AAPS. Compared with IFAS, the transmission delay is improved by $1-\frac{m\times \left(1-{\left(1-\frac{1}{m}\right)}^{k}\right)}{{\left(1-\frac{1}{m}\right)}^{k}+m\times \left(1-{\left(1-\frac{1}{m}\right)}^{k}\right)}\;$under AAPS scheme. □

**Figure 17:**
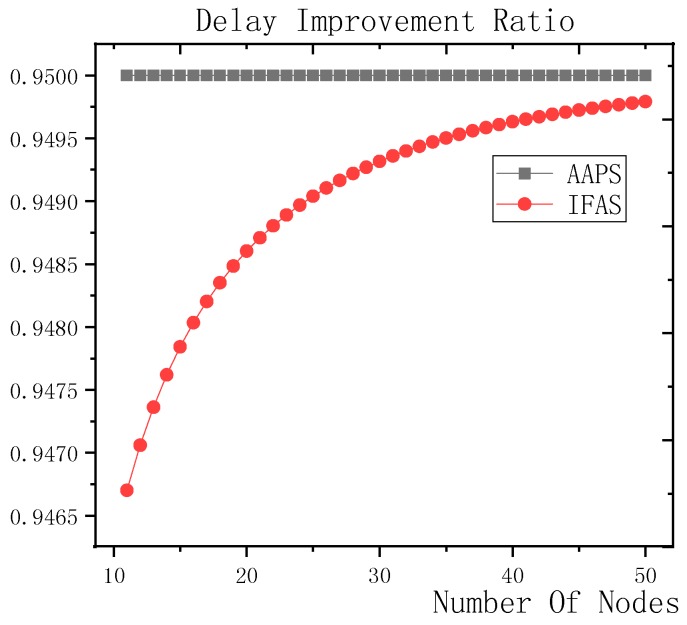
Comparison between IFAS algorithm and AAPS algorithm for transmission delay optimization (*m* = 20).

The improvement effect of IFAS and AAPS compared with traditional algorithm is shown in Figure 17. Since the slots added in AAPS algorithm promise minimum transmission delay, when the total number of slots in a cycle is constant, the improvement effect of AAPS is also constant as shown in the figure. While the improvement effect of IFAS increases as the number of son nodes increases, which is because, when there are more son nodes, the coverage of cycle is larger and the distribution is closer to the case of minimum transmission delay described in Theorem 5. Therefore, the improvement effect of IFAS gets closer to that of AAPS.

## 6. Experiment Results and Performances Comparison

In the experiment, we use a randomly generated tree to compare the performances of four algorithms in delay, transmission times and energy consumption. The tree has one sink node and 100 source nodes.

The experiment compares the performances of the four algorithms when the number of slots ($Num_{slot}$), single transmission success rate ($P_{trans}$) and total success rate threshold ($P_{th}$) changes. The parameter settings are shown in Table 6, Table 7 and Table 8 respectively.

When using the AAPS method in practice, the number of slots that each node can add needs calculation before it can be given. In the experiment, it is determined only by how many number of hops the nodes are away from the root node. When the number of hops is 2 or 3, only one slot can be added; when the number of hops is greater than 3, two slots can be added.

### 6.1. Diffusion Speed Comparison

**Figure 18:**
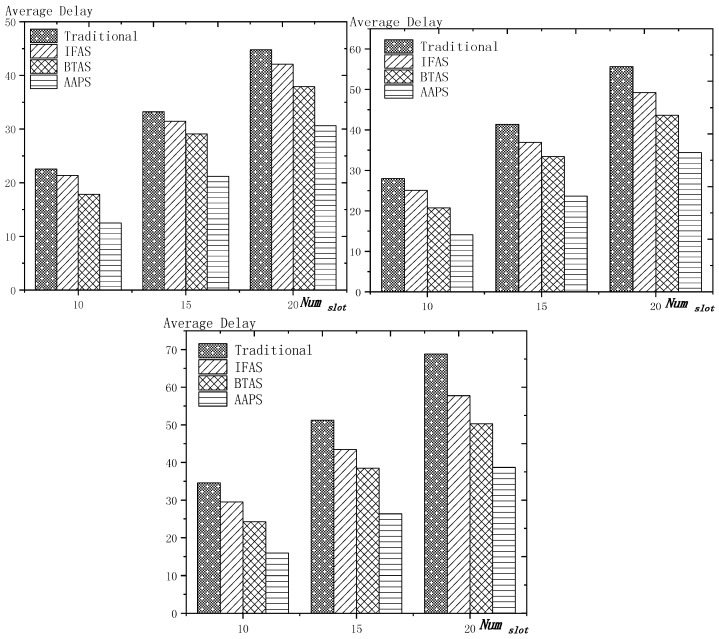
The average delay with $Num_{slot}$ as variable (setting 1, setting 2, setting 3).

As shown in Figure 18 is the average delay of the four algorithms with $Num_{slot}$ as variable. The values of the other parameters are set as described before. The abscissa is the number of slots and the ordinate is the average delay. When the number of slots is increased, the delay of the four algorithms is increased, which is because: for the traditional algorithm, after the first reception failure, it has to wait for one cycle and the increase of slots makes the cycle longer and extends the transmission delay. For the three algorithms proposed in this paper, as proved by Theorem 7 in Section 5.2, when the number of nodes remains unchanged and the number of slots increases, the slot coverage decreases, which makes the waiting time for nodes to receive for another time after failure longer. Therefore, the transmission delay is prolonged. Furthermore, due to the decrease of slot coverage, the empty delay is also increased. The improvement effect of the three algorithms compared with the traditional algorithm is shown in Table 9. The optimization effect of IFAS is 5.29–16.08%, that of BTAS is 12.39–29.89% and that of AAPS is 31.60–53.89%. The order of delay optimization ability of the three algorithms is consistent with the proof in Section 5.2. However, the magnitude of optimization does not reach the effect in Theorem 13 in Section 5.2. This is because: for the IFAS algorithm, the improving effect is affected by slot distribution. When slots are denser at the back of the cycle, the improving effect is poor. At this point, the last node is optimized by using BTAS algorithm and the optimization effect is twice as good as IFAS. As for the AAPS algorithm, the optimization effect is greatly reduced in comparison with ideal case because the number of added slots is far too small to reach the ideal coverage in the theoretical analysis. However, even so, the optimization effect is still considerable, with a minimum of 31.60% and a maximum of 53.89%.

**Figure 19:**
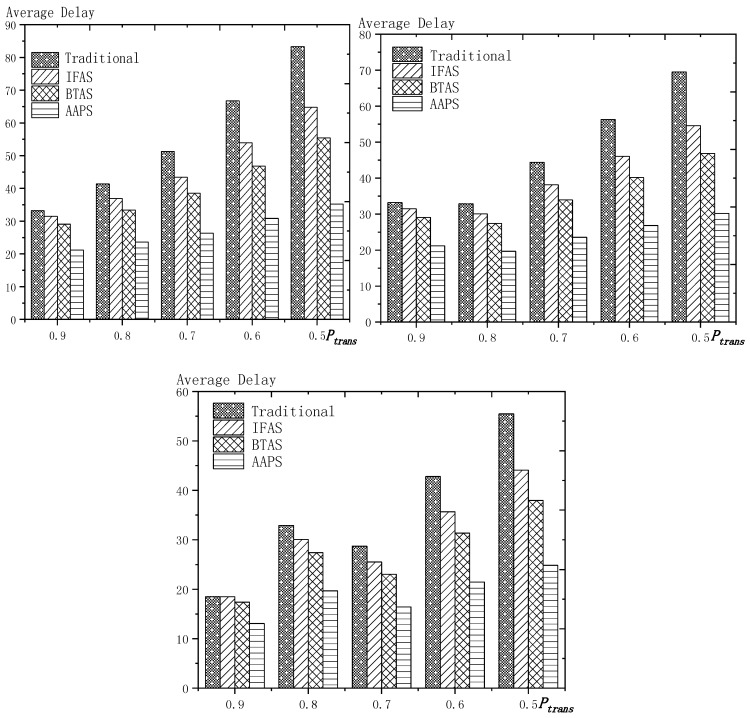
The average delay with $P_{trans}$ as variable (setting 1, setting 2, setting 3).

As shown in Figure 19 is the average delay of the four algorithms with $P_{trans}$ as variable. The values of the other parameters are set as described before. The abscissa is the single transmission success rate and the ordinate is the average delay. When the single transmission success rate decreases, the delay increases, which is because, as proved by Theorem 1 in Section 5.1, the single transmission success rate decreases, leading to increased retransmission times, thus increasing transmission delay. The optimization effect of the three algorithms proposed in this paper compared with the traditional algorithms is shown in Table 10. The optimization effect of IFAS algorithm is 0.00–22.25%, that of BTAS algorithm is 5.94–33.48% and that of AAPS is 29.35–57.72%. The minimum values are obtained when the single transmission success rate is 0.9 and the transmission success rate threshold is 0.9 and only one transmission is required. As shown in Theorem 9 in Section 5.2, the IFAS algorithm is equivalent to the traditional algorithm in this case. However, the BTAS algorithm still gets a certain degree of optimization because it optimizes the last node. Because the AAPS algorithm adds slots and reduces the empty delay, it can obtain 29.35% optimization effect when the IFAS algorithm fails to achieve optimization effect.

**Figure 20:**
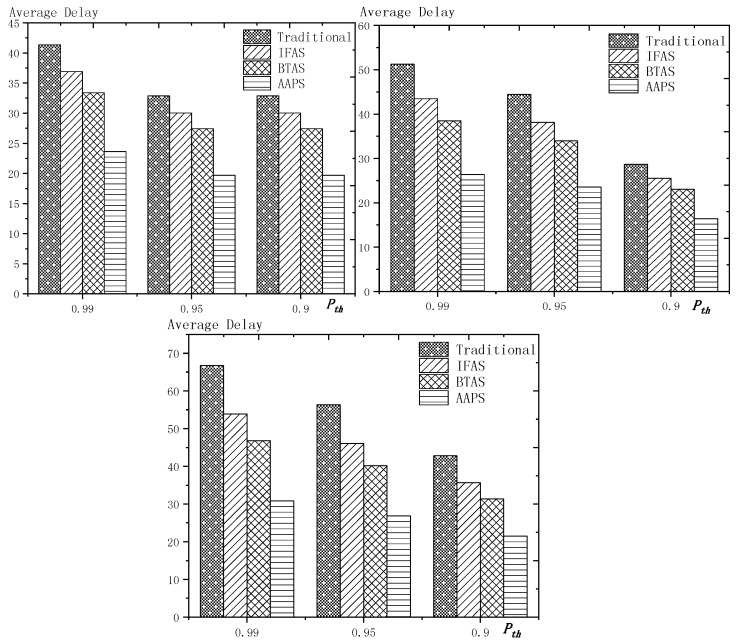
The average delay with $P_{th}$ as variable (setting 1, setting 2, setting 3).

As shown in Figure 20 is the average delay of the four algorithms with $P_{th}$ as variable. The values of the other parameters are set as described before. The abscissa is the total transmission success rate and the ordinate is the average delay. When the threshold value of the total transmission success rate decreases, the delay decreases, because: when the total transmission success rate decreases, the transmission times decreases and the transmission delay is shortened. Compared with the traditional algorithm, the optimization effect of the three algorithms proposed in this paper is shown in Table 11. The optimization effect of IFAS is 8.61–19.24%, that of BTAS is 16.60–29.81% and that of AAPS is 40.13–53.77%.

In terms of improving the performance of the diffusion rate, compared with traditional algorithm, the AAPS algorithm is increased by 31.60–55.16% and the BTAS algorithm is increased by 12.39–31.59% and the IFAS algorithm is increased by 0.00–20.56%. The minimum increase is obtained on the assumption that the packet can be successfully received with one send in the networks. Under this condition, the IFAS algorithm cannot make use of other slots after failure, which is identical to the traditional algorithm, while the BTAS algorithm and AAPS algorithm can still improve the performance due to the increase of awake slots. The maximum improvement is obtained in the case of poor networks conditions, that is, the single transmission success rate is very low and the packet needs multiple retransmission. The three algorithms proposed in this paper can significantly improve the performance of the networks in the aspect of delay when the networks condition is poor. This is because all three algorithms can reduce the transmission delay when multiple retransmission is required, whereas in the traditional algorithm, it takes one cycle to receive the code again when the node reception fails.

### 6.2. Transmission Times and Energy Consumption Comparison

**Figure 21:**
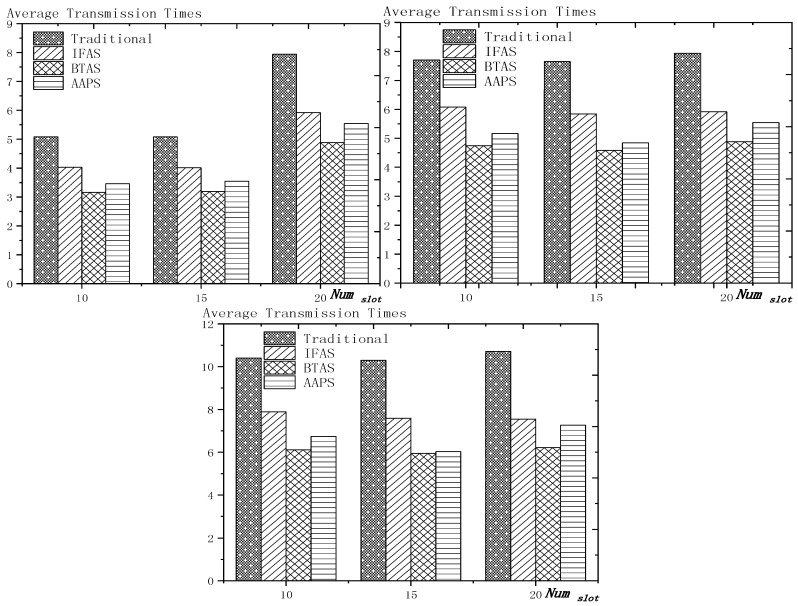
The average transmission times with $Num_{slot}$ as variable (setting 1, setting 2, setting 3).

As shown in Figure 21 is the average transmission times of the four algorithms with $Num_{slot}$ as variable. The values of the other parameters are set as described before. The abscissa is the number of slots and the ordinate is the average transmission times. When the number of slots increases, the number of transmission is almost unchanged or increases slightly. This is because, although the number of slots is increased, the number of nodes remains the same and because the single transmission success rate and total transmission success rate is unchanged, the number of transmission to a single node remains the same, so the total transmission number remains the same. However, as the number of slots increases, the slot coverage decreases and the number of slots shared by the nodes decreases, that is, the number of sons that wake up to receive the code from parent node at the same slot decreases, thus causing a slight increase in the transmission times. The improvement effect of the three algorithms compared with the traditional algorithm is shown in Table 12. The optimization effect of IFAS is 20.73–29.53%, that of BTAS is 37.24–42.26% and that of AAPS is 30.19–41.48%. All three algorithms can optimize because all three allow failed nodes to take advantage of the slots of other nodes. The optimization effect of the BTAS algorithm is the best, because the last node in the IFAS algorithm is always unable to share the time slot with other nodes and BTAS improves on this; in the AAPS algorithm, due to the increase of slot, every node can complete the reception of data packets quickly. Therefore, the node that wakes up earlier is less likely to share slot with the node that wakes later, resulting in fewer nodes that wake up at the same slot, so the parent node sends for more times than using BTAS.

**Figure 22:**
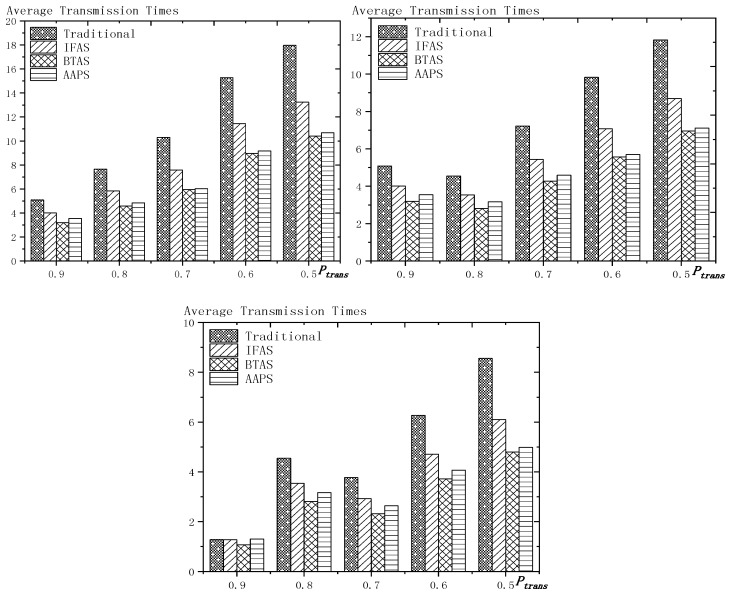
The average transmission times with $P_{trans}$ as variable (setting 1, setting 2, setting 3).

As shown in Figure 22 is the average transmission times of the four algorithms with $P_{trans}$ as variable. The values of the other parameters are set as described before. The abscissa is the single transmission success rate and the ordinate is the average transmission times. The improvement effect of the three algorithms compared with the traditional algorithm is shown in Table 13. The optimization effect of IFAS is 0.00–28.72%, that of BTAS is 16.09–43.93% and that of AAPS is −2.29–42.04%. When the total transmission success rate is 0.9 and the single transmission success rate is 0.9, only one transmission is required. In this case, the IFAS algorithm is equivalent to the traditional algorithm, so the optimization effect of transmission times is 0.00%. In other cases, the optimization effect of IFAS is always above 20%. When only one transmission is required, AAPS needs a bit more transmission times than traditional algorithm, which is because the slots are not distributed uniformly and the code can be sent at the slots when many nodes wake up or at other slots when only one node wakes up. Therefore, it is unknown whether the transmission times increases or decreases. However, the magnitude of increase or decrease is quite small. In this experiment, it only increases by 2.29%. In other cases, at least 29.98% optimization is obtained.

**Figure 23:**
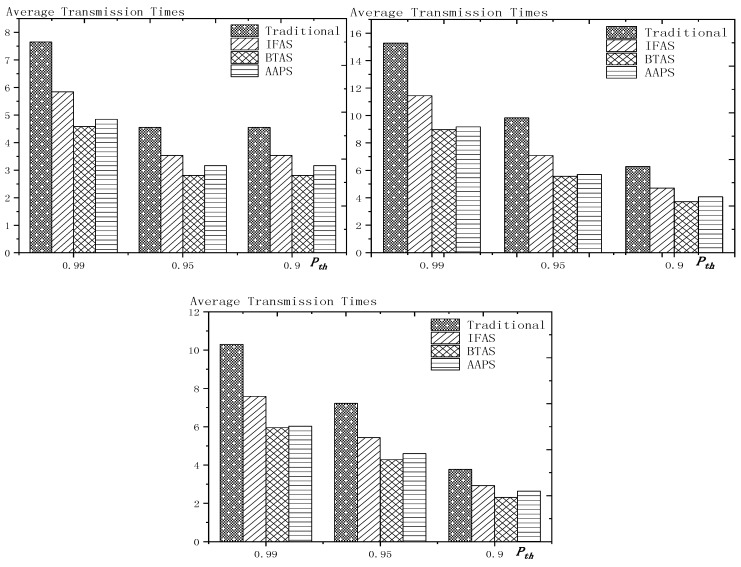
The average transmission times with $P_{th}$ as variable (setting 1, setting 2, setting 3).

As shown in Figure 23 is the average transmission times of the four algorithms with $P_{th}$ as variable. The values of the other parameters are set as described before. The abscissa is the total transmission success rate and the ordinate is the average transmission times. When the total transmission success rate decreases, the average transmission times decreases. This is because, as total transmission success rate decreases, so does the number of transmissions required by a single node and thus the total number of transmission also decreases. The improvement effect of the three algorithms compared with the traditional algorithm is shown in Table 14. The optimization effect of IFAS is 22.18–27.95%, that of BTAS is 38.20–43.39% and that of AAPS is 30.38–42.04%.

**Figure 24:**
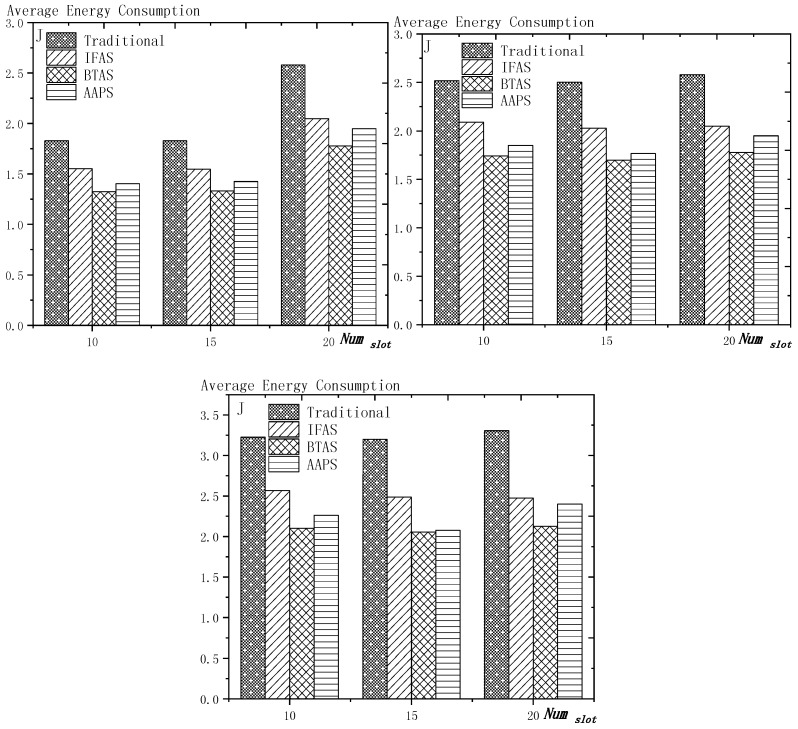
The average energy consumption with $Num_{slot}$ as variable (setting 1, setting 2, setting 3).

As shown in Figure 24 is the average energy consumption of the four algorithms with $Num_{slot}$ as variable. The values of the other parameters are set as described before. The abscissa is the number of slots and the ordinate is the average energy consumption. When the number of slots is increased, the average energy consumption is almost unchanged or slightly increases, which is the same case as the average transmission times. This is because the total energy consumption is mainly composed of the energy consumption of sending code and receiving code. The improvement effect of the three algorithms compared with the traditional algorithm is shown in Table 15. The optimization effect of IFAS is 15.12–25.10%, that of BTAS is 27.16–35.71% and that of AAPS is 22.05–35.04%. As analyzed above, the energy consumption during broadcasting is mainly composed of the energy consumed by the parent node’s sending the packet and the son nodes’ receiving the packet, so it is similar to the average transmission times.

**Figure 25:**
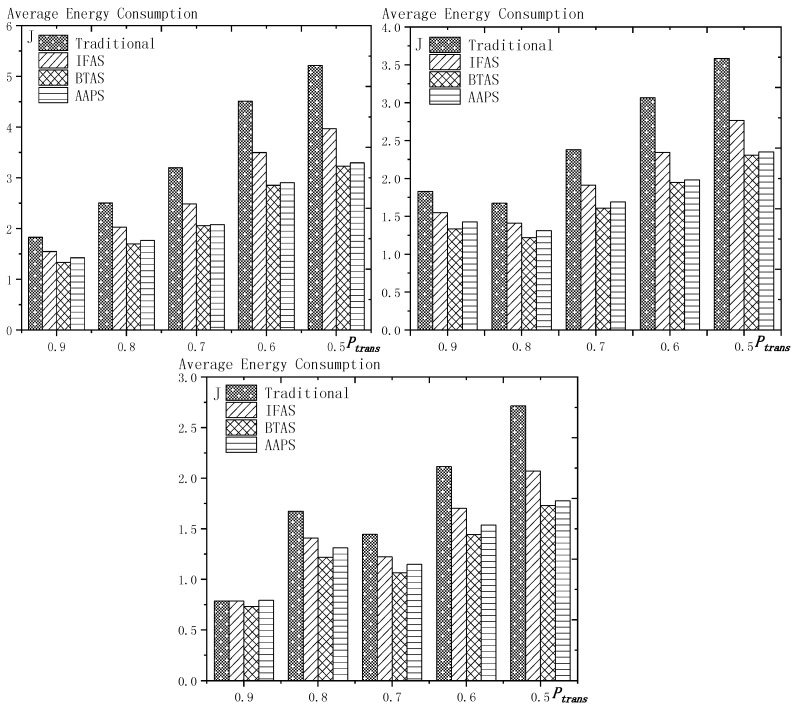
The average energy consumption with $P_{trans}$ as variable (setting 1, setting 2, setting 3).

As shown in Figure 25 is the average energy consumption of the four algorithms with $P_{trans}$ as variable. The values of the other parameters are set as described before. The abscissa is the single transmission success rate and the ordinate is the average transmission times. This is because, when the success rate of single transmission decreases, the number of retransmission increases. Therefore, the average energy consumption increases. The improvement effect of the three algorithms compared with the traditional algorithm is shown in Table 16. The optimization effect of IFAS is 0.00–23.86%, that of BTAS is 6.86–38.07% and that of AAPS is −0.97–36.75%. When the single transmission success rate is 0.9 and the threshold of the total transmission success rate is 0.9, only one transmission is required. In this case, IFAS is the same as the traditional algorithm, so the average energy consumption is not increased. AAPS, when code is transmitted only once, increased the energy consumption slightly by 0.97% due to a slight increase in transmission times. Since the nodes near the sink, which consumes the most energy, do not add slots, the lifetime of the networks will not be reduced.

**Figure 26:**
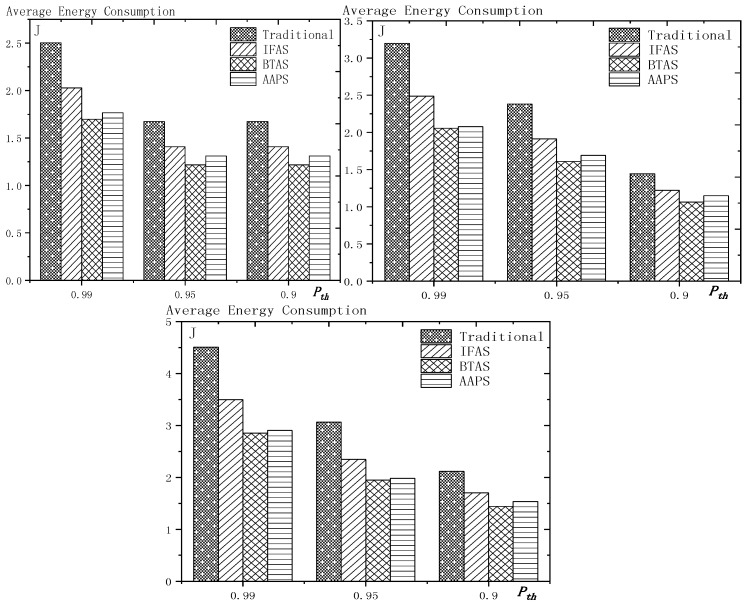
The average energy consumption with $P_{th}$ as variable (setting 1, setting 2, setting 3).

As shown in Figure 26 is the average energy consumption of the four algorithms with $P_{th}$ as variable. The values of the other parameters are set as described before. The abscissa is the total transmission success rate and the ordinate is the average energy consumption. When the total transmission success rate decreases, the average energy consumption decreases. This is because, as total transmission success rate decreases, the number of transmissions required by a single node decreases and thus the total number of transmission also decreases. The improvement effect of the three algorithms compared with the traditional algorithm is shown in Table 17. The optimization effect of IFAS is 15.45–23.50%, that of BTAS is 26.44–36.73% and that of AAPS is 20.53–35.58%. Since the average energy consumption is mainly affected by the average transmission times, the results are consistent with the average transmission times.

In terms of the improvement of transmission times, compared with the traditional algorithm, the AAPS algorithm is improved by −2.29–42.04%, the BTAS algorithm is improved by 16.09–43.93% and the IFAS algorithm is improved by 0.00%–29.53%.

In terms of the improvement of energy consumption, compared with the traditional algorithm, the AAPS algorithm is improved by −0.97–36.75%, the BTAS algorithm is improved by 6.86–36.49% and the IFAS algorithm is improved by 0.00–25.10%.

The minimum improvement of transmission times and energy consumption is obtained under the assumption that networks conditions are good enough and transmission can be completed at one time. Because only one transmission is needed, IFAS algorithm is equivalent to the traditional algorithm. Since the last node shares slot with the first node, BTAS can reduce the transmission times, energy consumption when IFAS fails to improve. Since the slots added are decided by the original slot, the node density at one slot is not uniform, therefore, transmission times can be increased or decreased. In the experiment, compared with the traditional algorithm, AAPS needs 2.29% more transmission times and 0.97% more energy but gets 29.35% delay optimization. 

However, in actual use, the networks condition is almost impossible to be good enough for transmission to be successful at one time. Therefore, the three algorithms proposed in this paper can achieve shorter delay in actual use with fewer transmission times and less energy consumption compared with the traditional algorithm.

## 7. Conclusions

In this paper, we proposed three broadcasting algorithms in WSNs. In previous research, a tradeoff was achieved instead of all-round optimization in delay, transmission times and energy consumption while maintaining long lifetime. By exploiting the broadcasting nature of wireless communication, the methods we designed made breakthrough. The theoretical analysis we did proved our methods practical and useful. The simulation result showed that IFAS, BTAS and AAPS respectively reduced delay by 20.56%, 31.59% and 55.16% and reduced transmission times by 29.53%, 43.93% and 42.04%. In most cases, IFAS achieved better results than traditional algorithm or the same results as it. BTAS under any circumstances obtained better results than traditional algorithm, AAPS got great optimization in terms of delay but in rare case (that is, in the case of non-failure transmission), led to a small increase in energy consumption, however, since the energy was consumed in area with energy surplus, lifetime was not shortened.

## Figures and Tables

**Table 1:** Symbols in the networks.

Symbol	Meaning	Value
m	the number of slots in a cycle	decided when computing
n	the number of source nodes in the networks	decided when computing
$P_{trans}$	possibility of successful single transmission	decided when computing
$P_{th}$	total successful transmission possibility threshold	decided when computing
$T_{max}$	maximum transmission times	decided when computing
$E_{trans}$	the energy required to send a packet	0.5 J
$E_{receive}$	the energy required to receive a packet	0.4 J
$E_{awake}$	the energy required when node wakes up to detect whether it needs to receive the packet	0.1 J

**Table 2:** Delay under traditional scheme.

$\;v_{1}$	$\;v_{2}$	$\;v_{3}$	$\;v_{4}$	$\;v_{5}$	$\;v_{6}$	$\;v_{7}$	$\;v_{8}$	$\;v_{9}$	$\;v_{10}$
16	12	23	40	28	39	32	28	40	36
$\;v_{11}$	$\;v_{12}$	$\;v_{13}$	$\;v_{14}$	$\;v_{15}$	$\;v_{16}$	$\;v_{17}$	$\;v_{18}$	$\;v_{19}$	$\;v_{20}$
47	64	52	63	48	44	47	56	52	56
$\;v_{21}$	$\;v_{22}$	$\;v_{23}$	$\;v_{24}$	$\;v_{25}$	$\;v_{26}$	$\;v_{27}$	$\;v_{28}$	$\;v_{29}$	$\;v_{30}$
44	48	44	64	52	56	52	64	60	71

**Table 3:** Delay under IFAS scheme.

$\;v_{1}$	$\;v_{2}$	$\;v_{3}$	$\;v_{4}$	$\;v_{5}$	$\;v_{6}$	$\;v_{7}$	$\;v_{8}$	$\;v_{9}$	$\;v_{10}$
7	7	23	15	15	31	16	20	31	31
$\;v_{11}$	$\;v_{12}$	$\;v_{13}$	$\;v_{14}$	$\;v_{15}$	$\;v_{16}$	$\;v_{17}$	$\;v_{18}$	$\;v_{19}$	$\;v_{20}$
47	23	23	39	23	23	39	40	44	32
$\;v_{21}$	$\;v_{22}$	$\;v_{23}$	$\;v_{24}$	$\;v_{25}$	$\;v_{26}$	$\;v_{27}$	$\;v_{28}$	$\;v_{29}$	$\;v_{30}$
28	32	36	40	44	40	44	55	55	71

**Table 4:** Delay under BTAS scheme.

$\;v_{1}$	$\;v_{2}$	$\;v_{3}$	$\;v_{4}$	$\;v_{5}$	$\;v_{6}$	$\;v_{7}$	$\;v_{8}$	$\;v_{9}$	$\;v_{10}$
7	7	7	15	15	15	24	28	15	15
$\;v_{11}$	$\;v_{12}$	$\;v_{13}$	$\;v_{14}$	$\;v_{15}$	$\;v_{16}$	$\;v_{17}$	$\;v_{18}$	$\;v_{19}$	$\;v_{20}$
15	23	23	23	23	23	23	24	28	40
$\;v_{21}$	$\;v_{22}$	$\;v_{23}$	$\;v_{24}$	$\;v_{25}$	$\;v_{26}$	$\;v_{27}$	$\;v_{28}$	$\;v_{29}$	$\;v_{30}$
36	40	44	24	28	24	28	23	23	23

**Table 5:** Delay under AAPS scheme.

$\;v_{1}$	$\;v_{2}$	$\;v_{3}$	$\;v_{4}$	$\;v_{5}$	$\;v_{6}$	$\;v_{7}$	$\;v_{8}$	$\;v_{9}$	$\;v_{10}$
7	7	23	12	11	15	16	12	28	27
$\;v_{11}$	$\;v_{12}$	$\;v_{13}$	$\;v_{14}$	$v_{15}$	$\;v_{16}$	$\;v_{17}$	$\;v_{18}$	$\;v_{19}$	$\;v_{20}$
31	16	15	17	16	15	15	22	19	22
$\;v_{21}$	$\;v_{22}$	$\;v_{23}$	$\;v_{24}$	$\;v_{25}$	$\;v_{26}$	$\;v_{27}$	$\;v_{28}$	$\;v_{29}$	$\;v_{30}$
24	19	16	35	32	35	32	35	33	36

**Table 6:** Parameter settings with $Num_{slot}$ as variable.

Setting	$\;P_{th}\;$	$\;P_{trans}\;$
1	0.99	0.9
2	0.99	0.8
3	0.99	0.7

**Table 7:** Parameter settings with $P_{trans}$ as variable.

Setting	$\;Num_{slot}\;$	$\;P_{th}\;$
1	15	0.99
2	15	0.95
3	15	0.90

**Table 8:** Parameter settings with $P_{th}$ as variable.

Setting	$\;Num_{slot}\;$	$\;P_{trans}\;$
1	15	0.8
2	15	0.7
3	15	0.6

**Table 9:** The improvement of delay compared with the traditional algorithm.

$\;Num_{slot}\;$	IFAS (Setting 1, Setting 2, Setting 3)	BTAS (Setting 1, Setting 2, Setting 3)	AAPS (Setting 1, Setting 2, Setting 3)
10	5.36%, 10.43%, 14.56%	20.94%, 25.88%, 29.89%	44.51%, 49.64%, 53.89%
15	5.29%, 10.75%, 15.24%	12.39%, 19.24%, 24.88%	36.19%, 42.87%, 48.56%
20	6.01%, 11.55%, 16.08%	15.37%, 21.68%, 26.88%	31.60%, 38.20%, 43.74%

**Table 10:** The improvement of delay compared with the traditional algorithm.

$\;P_{trans}\;$	IFAS (Setting 1, Setting 2, Setting 3)	BTAS (Setting 1, Setting 2, Setting 3)	AAPS (Setting 1, Setting 2, Setting 3)
0.9	5.29%, 5.29%, 0.00%	12.39%, 12.39%, 5.94%	36.19%, 36.19%, 29.35%
0.8	10.75%, 8.61%, 8.61%	19.24%, 16.60%, 16.60%	42.87%, 40.13%, 40.13%
0.7	15.24%, 14.15%, 10.93%	24.88%, 23.55%, 19.62%	48.56%, 46.93%, 42.73%
0.6	19.24%, 18.25%, 16.69%	29.81%, 28.68%, 26.79%	53.77%, 52.32%, 49.87%
0.5	22.25%, 21.45%, 20.56%	33.48%, 32.54%, 31.59%	57.72%, 56.55%, 55.16%

**Table 11:** The improvement of delay compared with the traditional algorithm.

$\;P_{th}\;$	IFAS (Setting 1, Setting 2, Setting 3)	BTAS (Setting 1, Setting 2, Setting 3)	AAPS (Setting 1, Setting 2, Setting 3)
0.99	10.75%, 15.24%, 19.24%	19.24%, 24.88%, 29.81%	42.87%, 48.56%, 53.77%
0.95	8.61%, 14.45%, 18.25%	16.60%, 23.55%, 28.68%	40.13%, 46.93%, 52.32%
0.90	8.61%, 10.93%, 16.69%	16.60%, 19.62%, 26.79%	40.13%, 42.73%, 49.88%

**Table 12:** The improvement of transmission times compared with the traditional algorithm.

$\;Num_{slot}\;$	IFAS (Setting 1, Setting 2, Setting 3)	BTAS (Setting 1, Setting 2, Setting 3)	AAPS (Setting 1, Setting 2, Setting 3)
10	20.73%, 21.06%, 24.16%	37.85%, 38.39%, 41.21%	31.94%, 32.97%, 35.25%
15	21.13%, 23.66%, 26.33%	37.24%, 40.12%, 42.26%	30.23%, 36.68%, 41.48%
20	25.45%, 25.45%, 29.53%	38.50%, 38.50%, 41.93%	30.19%, 30.19%, 32.15%

**Table 13:** The improvement of transmission times compared with the traditional algorithm.

$\;P_{trans}\;$	IFAS (Setting 1, Setting 2, Setting 3)	BTAS (Setting 1, Setting 2, Setting 3)	AAPS (Setting 1, Setting 2, Setting 3)
0.9	21.11%, 21.11%, 0.00%	37.24%, 37.24%, 16.09%	30.23%, 30.23%, −2.29%
0.8	23.66%, 22.18%, 22.18%	40.12%, 38.20%, 38.20%	36.68%, 30.38%, 30.38%
0.7	26.33%, 24.73%, 22.55%	42.26%, 40.76%, 38.59%	41.48%, 36.36%, 29.97%
0.6	25.16%, 27.95%, 24.95%	41.29%, 43.39%, 40.76%	40.00%, 42.04%, 35.14%
0.5	26.37%, 26.47%, 28.72%	42.08%, 41.20%, 43.93%	40.61%, 39.83%, 41.77%

**Table 14:** The improvement of transmission times compared with the traditional algorithm.

$\;P_{th}\;$	IFAS (Setting 1, Setting 2, Setting 3)	BTAS (Setting 1, Setting 2, Setting 3)	AAPS (Setting 1, Setting 2, Setting 3)
0.99	23.66%, 26.33%, 25.16%	40.12%, 42.26%, 41.29%	36.68%, 41.48%, 40.00%
0.95	22.18%, 24.73%, 27.95%	38.20%, 40.76%, 43.39%	30.38%, 36.36%, 42.04%
0.90	22.18%, 22.55%, 24.95%	38.20%, 38.59%, 40.76%	30.38%, 29.97%, 35.14%

**Table 15:** The improvement of energy consumption compared with the traditional algorithm.

$\;Num_{slot}\;$	IFAS (Setting 1, Setting 2, Setting 3)	BTAS (Setting 1, Setting 2, Setting 3)	AAPS (Setting 1, Setting 2, Setting 3)
10	15.12%, 16.91%, 20.44%	27.61%, 30.83%, 34.87%	23.29%, 26.47%, 29.83%
15	15.40%, 18.97%, 22.25%	27.16%, 32.17%, 35.71%	22.05%, 29.41%, 35.04%
20	20.55%, 20.55%, 25.10%	31.09%, 31.09%, 35.64%	24.38%, 24.38%, 27.33%

**Table 16:** The improvement of energy consumption compared with the traditional algorithm.

$\;P_{trans}\;$	IFAS (Setting 1, Setting 2, Setting 3)	BTAS (Setting 1, Setting 2, Setting 3)	AAPS (Setting 1, Setting 2, Setting 3)
0.9	15.40%, 15.40%, 0.00%	27.16%, 27.16%, 6.86%	22.05%, 22.05%, −0.97%
0.8	18.97%, 15.82%, 15.82%	32.17%, 27.24%, 27.24%	29.41%, 21.67%, 21.67%
0.7	22.25%, 19.67%, 15.45%	35.71%, 32.43%, 26.44%	35.04%, 28.93%, 20.53%
0.6	22.38%, 23.50%, 19.43%	36.73%, 36.49%, 31.74%	35.58%, 35.36%, 27.36%
0.5	23.86%, 22.89%, 23.76%	38.07%, 35.64%, 36.34%	36.75%, 34.45%, 34.56%

**Table 17:** The improvement of energy consumption compared with the traditional algorithm.

$\;P_{th}\;$	IFAS (Setting 1, Setting 2, Setting 3)	BTAS (Setting 1, Setting 2, Setting 3)	AAPS (Setting 1, Setting 2, Setting 3)
0.99	18.97%, 22.25%, 22.38%	32.17%, 35.71%, 36.73%	29.41%, 35.04%, 35.58%
0.95	15.82%, 19.67%, 23.50%	27.24%, 32.43%, 36.49%	21.67%, 28.93%, 35.36%
0.90	15.82%, 15.45%, 19.43%	27.24%, 26.44%, 31.74%	21.67%, 20.53%, 27.36%
